# APP Processing Induced by Herpes Simplex Virus Type 1 (HSV-1) Yields Several APP Fragments in Human and Rat Neuronal Cells

**DOI:** 10.1371/journal.pone.0013989

**Published:** 2010-11-15

**Authors:** Giovanna De Chiara, Maria Elena Marcocci, Livia Civitelli, Rafaela Argnani, Roberto Piacentini, Cristian Ripoli, Roberto Manservigi, Claudio Grassi, Enrico Garaci, Anna Teresa Palamara

**Affiliations:** 1 Department of Cell Biology and Neuroscience, Istituto Superiore di Sanità, Rome, Italy; 2 Department of Public Health and Infectious Diseases, Sapienza University of Rome, Rome, Italy; 3 Department of Experimental Medicine and Diagnostic, University of Ferrara, Ferrara, Italy; 4 Institute of Human Physiology, Catholic University Medical School, Rome, Italy; 5 Department of Experimental Medicine and Biochemical Sciences, University of Rome Tor Vergata, Rome, Italy; 6 Department of Public Health and Infectious Diseases, Institute Pasteur Cenci Bolognetti Foundation, Sapienza University of Rome, Rome, Italy; 7 San Raffaele Pisana Scientific Institute for Research, Hospitalization and Health Care, Rome, Italy; Mount Sinai School of Medicine, United States of America

## Abstract

Lifelong latent infections of the trigeminal ganglion by the neurotropic herpes simplex virus type 1 (HSV-1) are characterized by periodic reactivation. During these episodes, newly produced virions may also reach the central nervous system (CNS), causing productive but generally asymptomatic infections. Epidemiological and experimental findings suggest that HSV-1 might contribute to the pathogenesis of Alzheimer's disease (AD). This multifactorial neurodegenerative disorder is related to an overproduction of amyloid beta (Aβ) and other neurotoxic peptides, which occurs during amyloidogenic endoproteolytic processing of the transmembrane amyloid precursor protein (APP). The aim of our study was to identify the effects of productive HSV-1 infection on APP processing in neuronal cells. We found that infection of SH-SY5Y human neuroblastoma cells and rat cortical neurons is followed by multiple cleavages of APP, which result in the intra- and/or extra-cellular accumulation of various neurotoxic species. These include: i) APP fragments (APP-Fs) of 35 and 45 kDa (APP-F35 and APP-F45) that comprise portions of Aβ; ii) N-terminal APP-Fs that are secreted; iii) intracellular C-terminal APP-Fs; and iv) Aβ_1-40_ and Aβ_1-42_. Western blot analysis of infected-cell lysates treated with formic acid suggests that APP-F35 may be an Aβ oligomer. The multiple cleavages of APP that occur in infected cells are produced in part by known components of the amyloidogenic APP processing pathway, i.e., host-cell β-secretase, γ-secretase, and caspase-3-like enzymes. These findings demonstrate that HSV-1 infection of neuronal cells can generate multiple APP fragments with well-documented neurotoxic potentials. It is tempting to speculate that intra- and extracellular accumulation of these species in the CNS resulting from repeated HSV-1 reactivation could, in the presence of other risk factors, play a co-factorial role in the development of AD.

## Introduction

Herpes simplex virus type 1 (HSV-1) is a ubiquitous neurotropic DNA virus that typically causes recurrent blister-like lesions on and around the lips in humans. However, it can also cause keratitis, as well as a rare form of encephalitis [1]. HSV-1 has been found in a latent form in the brains of a high proportion of elderly individuals [2], [3]. Primary HSV-1 infection is often followed by the establishment of latent infection in the peripheral nervous system (PNS), usually in the trigeminal ganglia. Reactivation, which may occur periodically, is followed by axonal transport of newly produced HSV-1 virions back to the site of the primary infection, where they cause new skin vesicles or mucosal ulcers. The reactivated virus can also move upward to the central nervous system (CNS), where it can cause a productive, but usually mild infection, that can later becomes latent [4]–[7].

Ball et al. [8] pointed out that the brain regions most frequently involved in herpes encephalitis are also the earliest and most severely involved targets of the neurodegenerative alterations of Alzheimer's disease (AD), a multifactorial disorder characterized by severe memory impairment and cognitive decline [9]. Possible links between AD and HSV-1 infection have also emerged from epidemiologic studies. The HSV-1 genome has reportedly been found in post-mortem brain specimens from many AD patients [4], [10]–[13], particularly those that carry the type 4 allele of the gene that encodes apolipoprotein E [14]. More recently, a large prospective population-based study also showed that the risk of AD is increased in elderly subjects with positive titers of anti-HSV-1 IgM antibodies, which are markers of primary or reactivated HSV-1 infection [15]. This observation supports the view that repeated reactivation of this virus may contribute to the development of AD.

One of the most widely accepted hypotheses on the molecular pathogenesis of AD focuses on the overproduction of amyloid beta (Aβ) peptides by neurons. The accumulation of these peptides in the extracellular spaces gives rise to the amyloid plaques that are one of the main neuropathological features of AD. Amyloid β is produced by endoproteolysis of a transmembrane glycoprotein known as the amyloid precursor protein (APP). APP can be processed along two different pathways. The first (which is physiologic) involves sequential proteolytic cleavages by the α- and γ-secretases, and it yields fragments that are generally recognized to be nontoxic (e.g., the soluble N-terminal fragment, a short peptide known as p3). Amyloid β is generated by an alternative form of APP processing, which begins when the parental protein undergoes cleavage by the β-secretase (also known as β-site APP cleaving enzyme 1 or BACE1) [16], [17]. This cut yields two species, the large N-terminal ectodomain of the precursor and the 99-amino-acid C-terminus stub. Subsequent cleavage of the latter fragment (between residues 38 and 43) by the γ-secretase complex results in the formation of Aβ species, which contain 40–42 amino acids, and the APP intracellular domain (AICD), whose ability to modulate gene expression, apoptosis, and cytoskeletal dynamics has also been linked to AD [18]. Amyloidogenic cleavage of APP is not confined to the cell membrane: it can also take place in the intermediate compartment of the endoplasmic reticulum [19]–[21], the trans-Golgi network [22], and the endosomal/lysosomal system [23]. In these cases, the Aβ species produced may be secreted into the extracellular space, where their build-up can promote senile plaque formation, or they may remain within the cell, where their aggregation can trigger neurotoxic processes and contribute to the progression of AD [24]. The APP can also be cleaved by other proteases. Cuts produced by caspases 3, 6, 7, and 8, for example, yield a C31 fragment of the protein that has also been implicated in the pathogenesis of AD [25], [26].

Several lines of experimental evidence point to possible links between HSV-1 and Aβ. Amyloid β is characterized by some degree of sequence homology with the HSV-1 glycoprotein B, and the viral protein has been suggested by some to act as a seed for Aβ deposition in amyloid plaques [27]. Other investigators [28] have hypothesized that new HSV-1 particles produced in the PNS recruit cell membranes containing APP, possibly during packaging in the Golgi apparatus. Transport of infective virus to the brain might then be followed by the release and hydrolysis of APP, which might contribute in some way to the formation of amyloid deposits. These researchers have also suggested that the APP itself plays a role in the transport of HSV-1 into neurons [28]–[30]. Wozniack et al. [31] reported the accumulation of Aβ peptides in neurons and mouse brains infected with HSV-1, and they also demonstrated the presence of the viral genome within amyloid plaques in AD brains. Other studies suggest that HSV-1 infection can interfere with APP processing. Shipley et al. [32] found that HSV-1 infection of neuroblastoma cells led to the appearance of a 55-kDa C-terminal fragment of APP, and Wozniack et al. [33] found that BACE1 (β-secretase) and nicastrin (an essential component of γ-secretase complex) immunolabeling is increased in the brains of HSV-1-infected mice. We recently reported that HSV-1 produces marked changes in neuronal excitability and intracellular Ca^2+^ signalling that cause APP phosphorylation and intracellular Aβ accumulation in rat cortical neurons [34].

Taken together, these findings prompted us to investigate the role of HSV-1 in promoting the formation in neurons of multiple neurotoxic APP fragments that can contribute to the development of AD. To address this issue, we used a multi-pronged approach to characterize the effects of this virus on APP processing in neuronal cells. We demonstrate here that HSV-1 triggers amyloidogenic cleavages of the APP that are mediated in part by the action of β-secretase, γ-secretase, and caspase-3-like enzymes, and that result in the formation and intracellular accumulation of different APP fragments with established potential for neurotoxicity.

## Materials and Methods

### Ethics Statement

All animal procedures were approved by the Ethics Committee of the Catholic University and complied with Italian Ministry of Health guidelines and with national laws (Legislative decree 116/1992) and European Union guidelines on animal research (No. 86/609/EEC).

Unless otherwise stated, all commercial products and devices mentioned below were used in accordance with manufacturers' instructions.

### Cell cultures

Human SH-SY5Y neuroblastoma and HeLa cells were grown in Dulbecco's modified Eagle's medium (Euroclone) containing 15% heat-inactivated fetal bovine serum (FBS, Gibco), glutamine (0.3 mg/ml), penicillin (100 units/ml), and streptomycin (100 µg/ml). VERO cells were maintained in RPMI 1640 medium (Gibco) supplemented with 10% FBS and antibiotics.

Primary cultures of cortical neurons were prepared from E17-E18 rat embryos according to standard protocols [35] with some modifications. Briefly, the brain cortex was dissected in sodium phosphate buffer solution (PBS) and digested with trypsin (0.025%) for 10 min at 37°C. The digestion was stopped by the addition of 5% FBS. Cells were triturated through a fire-polished Pasteur pipette to obtain a single-cell suspension and plated onto poly-L-lysine-treated dishes at a density of 1×10^6^ cells per dish. Cultured neurons were used for viral infection 8 days after plating.

### Virus production, infection, and titration

Monolayers of VERO cells in 75-cm^2^ tissue culture flasks were infected with HSV-1 strain F at a multiplicity of infection (m.o.i.) of 0.01. After 48 hours at 37°C, infected cells were harvested with 3 freeze-and-thaw cycles. Cells were removed with low-speed centrifugation, and virus titers were measured by standard plaque assay [36]. The titer of the virus preparation was 4×10^9^ plaque-forming units (pfu) per ml.

Twenty-four hours after SH-SY5Y plating (or 8 days after cortical neuron preparation), cells were challenged with wild-type or mutant HSV-1 (m.o.i 1) for 1 h at 37°C, washed with PBS, and then incubated with medium supplemented with 2% FBS. Mock-infection was performed with conditioned medium from uninfected VERO cells at the same dilution as that used for the virus. Virus production was assessed by standard plaque assay of cell supernatants collected at different times post infection (p.i.).

R3616 and R2621 mutant viruses were a kind gift from Dr. B. Roizman of the University of Chicago's Marjorie B. Kovler Viral Oncology Laboratories. They are HSV-1 strain F-based viruses deleted in both copies of the *UL34.5* and *vhs* genes, respectively.

For the experiments with inactivated viruses, HSV-1 was maintained at 70°C for 30 min (heat inactivated HSV-1) or exposed on ice for 5 min to a 30 W, 254 nm, germicidal UV light placed at a distance of 15 cm (UV-inactivated HSV-1). No viral particles were detected by plaque assay in the supernatants of cell cultures infected with UV- or heat-inactivated HSV-1. Heat-inactivated HSV-1 is incapable of binding to the plasma membrane and infect cells, whereas UV-inactivated HSV-1 retains the capacity for plasma membrane binding and cell entry although it is incapable of replication.

### Reagents

DAPI (4',6-diamidino-2-phenylindole dihydrochloride; Invitrogen) was diluted to 0.5 µg/ml in PBS. Stock solutions of γ-secretase inhibitor X and caspase inhibitor I (or Z-VAD; Calbiochem), both dissolved in DMSO, were diluted in DMEM to final concentrations of 1 µM and 50 µM, respectively. A stock solution of β-secretase inhibitor (Calbiochem) dissolved in PBS was diluted in DMEM to a final concentration of 1 µM. The highest DMSO concentration present in the culture medium was 0.2%. Control cells were treated with DMSO alone at the same concentration present in the test substance being evaluated. Cycloheximide (CHX, Sigma) was used at a final concentration of 50 µg/ml; phosphonacetic acid (PAA, Sigma) was used at a final concentration of 500 µg/ml. Mouse monoclonal antibodies raised against Aβ residues 17-24 (4G8), rabbit polyclonal antibodies against Aβ_1-42_, and mouse monoclonal antibodies against Aβ_1-40_ (11A50-B10) were purchased from Covance. Goat polyclonal anti-HSV-1, mouse monoclonal anti-APP A4 (MAB348, clone 22C11), and mouse monoclonal anti-APP 643-695 (MAB343, clone 2.F2.19B4) antibodies were purchased from Chemicon. Mouse monoclonal anti-actin and anti-tubulin were purchased from Sigma.

Antibodies to Aβ_1-10_ (M2°) were produced in-house by immunizing New Zealand White rabbits with a synthetic peptide containing residues 1-10 of the human Aβ peptide. The animals were bled 10 days after the last injection. The antiserum's recognition of Aβ was confirmed by western blot analyses. Aliquots were stored at −20°C.

### Luciferase assay

The pRC-CMV vector expressing the APP695-Gal4 fusion protein, the G5B-luc vector containing 5 five consecutive Gal4 binding sites upstream luciferase cDNA, and the phRL-CMV vector containing cDNA encoding Renilla luciferase were kind gifts of Prof. Tommaso Russo (CEINGE, Federico II University of Naples, Naples, Italy). Luciferase activity was measured as a readout of APP cleavage, as described elsewhere [37]. Briefly, HeLa cells were stably transfected with the pRC-CMV vector encoding APP695-Gal4 (HeLaAG cells) and transiently co-transfected with the G5B-luciferase vector, which expresses luciferase under the control of 5 Gal4-responsive elements. Twenty-four hours after transfection, these cells were infected with HSV-1 at an m.o.i. of 1. Luciferase activity was then measured at different times p.i. with the Dual Reporter Luciferase Assay System (Promega).

### Western blot analysis

Cells were washed with PBS, resuspended in cold lysis buffer (10 mM Tris-HCl, 150 mM NaCl, 1 mM phenylmethylsulfonyl fluoride (PMSF), phosphatase inhibitor mixture [Sigma], and 1% Triton X-100, pH 7.4), and incubated for 30 min on ice. After centrifugation (13000×*g* for 30 min) the supernatants were collected and assayed to determine their protein concentration (Bradford method, Bio-Rad). Equivalent amounts of proteins were resuspended in SDS sample buffer containing 10% β-mercaptoethanol, separated with SDS-PAGE, and blotted onto nitrocellulose membranes for western blot analysis. The membranes were blocked with 10% nonfat dry milk in PBS for 1 h at room temperature. Primary antibodies were used at a final concentration of 1 µg/ml. Secondary antibodies were horseradish peroxidase-conjugated (Jackson ImmunoResearch). Blots were developed with the ECL-Plus Detection System (GE Healthcare) and subjected to densitometric scanning. When necessary, membranes were stripped by heating at 56°C in 62.5 mM Tris-HCl, pH 6.7, with 100 mM 2-mercaptoethanol and 2% SDS.

### Immunoprecipitation studies

Nuclear and cytoplasmic extracts from mock- and HSV-1-infected SH-SY5Y cells were prepared with the NE-PER Nuclear and Cytoplasmic extraction kit (Pierce), and their protein concentrations were determined with the BCA Protein Assay kit (Pierce). Equal amounts of nuclear and cytoplasmic protein extracts were incubated with anti-APP MAB343 antibody (1 µg/ml) overnight at 4°C to immunoprecipitate APP C-terminal fragments. Immunoprecipitates were separated by 15% SDS-PAGE gel, blotted onto 0.22 µm nitrocellulose membrane and stained with anti-APP MAB343 antibody. Blots were developed as described above.

### Trichloroacetic acid protein precipitation

To investigate the production of soluble APP fragments that were secreted from infected cells, we collected equivalent amounts of medium conditioned by mock- and HSV-1-infected cells and incubated them overnight with 10% trichloroacetic acid (TCA) at 4°C. The mixture was centrifuged at 13000×*g* for 30 min. The protein pellet was washed twice with 1 ml of 80% ice-cold acetone and dissolved in basic solution containing 1 M Trizma base. The latter was accomplished by repeated up-and-down pipetting and vortexing every 10 min for 1 hour.

### Formic acid

Cells (2×10^6^ per sample) were washed with PBS and centrifuged at 700×*g* for 5 min. The pellet was lysed in 70% formic acid, in 10% SDS, or in standard lysis buffer containing 1% Triton-X, PMSF, and a protease-inhibitor cocktail (Sigma). After 30 min on ice, lysates were placed in an ice box and sonicated with a probe sonicator for 8 sec (4 times). Formic acid was evaporated under vacuum with a Centrivac (Heraeus Instruments), and 1 M Trizma base (FA fraction) was added to neutralize the sample. Lysates (Triton- and SDS-soluble fractions) were centrifuged at 13000×*g* for 30 min, and the supernatants were collected.

### Preparation of Aβ_1–42_ oligomers

Lyophilized Aβ_1–42_ peptide (Bachem) was dissolved in 100% 1,1,1,3,3,3-hexafluoro-2-propanol (HFIP, Sigma) (final concentration, 1 mM) to eliminate any aggregates that might be present. The HFIP was then removed by vacuum evaporation in a Centrivac (Heraeus Instruments). The dried film that remained was dissolved in dimethyl-sulfoxide (DMSO, final concentration 1 mM) and stored at -20°C. Immediately before use, the Aβ_1–42_-DMSO solution was diluted to a concentration of 100 µM in ice-cold cell culture medium (phenol red-free) and incubated at 4°C for 48 h to allow the formation of Aβ_1-42_ oligomers.

### Real-time PCR

Total RNA was isolated from mock- and HSV-1-infected SH-SY5Y cells harvested at the indicated times p.i. with the RNeasy Kit (Qiagen) and quantified by ultraviolet spectrometry at 260 nm. The iScript™ cDNA Synthesis Kit (Biorad) was used to reverse-transcribe 1 µg of RNA into cDNA in a final volume of 20 ml. Relative quantitative real-time PCR was performed in an iCycler IQ5 (Biorad) with the IQ™ SYBR Green Supermix (Biorad) reagent. Fluorescein was included in each reaction for well-factor collection. All PCR reactions were coupled to melting-curve analysis to confirm the amplification specificity. Primer sequences are shown in Table 1. After an initial denaturation step at 95°C for 3 min, PCR involved 40 cycles at 95°C for 10 sec and at 58°C for 30 sec. Quantification of each PCR product was expressed relative to rRNA 18S. The relative quantification was calculated with the analysis software provided with the iCycler IQ5 (Biorad).

**Table 1:** Nucleotide sequences of the primers used for real-time PCR.

APP	Forward: 5′-AGACTATGCTGATGGCGGTGAAG-3′
	Reverse: 5′-CAATGCTGGTTGTTCTCTCTGTGG-3′
rRNA 18S	Forward: 5′-GTAACCCGTTGAACCCCATT-3′
	Reverse: 5′-CCATCCAATCGGTAGTAGCG-3′

### Confocal immunofluorescence microscopy

SH-SY5Y cells were processed for confocal immunofluorescence microscopy according to a standard protocol [38]. Briefly, cells were fixed with 4% paraformaldehyde (Sigma) in PBS for 10 min at room temperature, rinsed twice in PBS, and permeabilized by 15 min incubation with PBS/Triton X-100 (0.3%, Sigma). Cells were then blocked for 20 min in 0.3% bovine serum albumin (in PBS) and incubated overnight at 4°C with different pairs of the following antibodies: MAB343 (1∶200), goat anti-HSV-1 (1∶500), rabbit anti-Aβ_1-42_ (1∶200), and mouse anti-Aβ_1-40_ (1∶200). The following day, cells were washed twice in PBS and then incubated for 90 min at room temperature with a mixture of the following secondary antibodies: goat anti-rabbit Alexa Fluor 488 (1∶1000; Invitrogen), donkey anti-goat Alexa Fluor 633 (1∶1000, Invitrogen), and rhodamine-conjugated goat anti-mouse (1∶300, Millipore). Nuclei were then counterstained for 10 min with DAPI, and the cells were coverslipped with ProLong Gold Antifade Reagent (Invitrogen). Images from at least 10 random 63× fields were acquired with a confocal laser scanning system (TCS-SP2, Leica Microsystem, GmbH) equipped with an Ar/ArKr laser for 488-nm excitation and two NeHe lasers for 543- and 633-nm excitation. DAPI staining was imaged by two-photon excitation (740 nm, <140 fs, 90 MHz) with an ultrafast, tunable, mode-locked titanium sapphire laser (Chamaleon, Coherent Inc.). All experiments were repeated at least 3 times. Nuclear C-terminus APP immunoreactivity was quantified as mean of fluorescence intensities measured in regions of interest (ROI) traced around every cell nucleus.

### ELISA

For quantification of HSV-1-induced Aβ_1-42_, we used APP695 transiently transfected SH-SY5Y. To this aim, cells were transfected by Lipofectamine 2000 (Life Technologies, Inc.) with 4 µg of APP695 plasmid (kindly provided by Tommaso Russo, CEINGE, Federico II University of Naples, Naples, Italy), according to the manufacturer's instructions. After 24 h, cells were infected with HSV-1 at 1 m.o.i for 18 h. Briefly, for intracellular Aβ_1-42_ quantification, SH-SY5Y cell pellets were dissolved in 70% formic acid and sonicated as previously described. Formic acid was evaporated under vacuum with a Centrivac (Heraeus Instruments), and 2 M Trizma base was added to neutralize the sample. Then sample were diluited 1∶3 in H_2_O before protein quantification with BCA Protein Assay Kit (Pierce) and quantification of Aβ_1-42_ by ELISA assay. For extracellular Aβ_1-42_ quantification, cell supernatants were centrifuged first at 10000×*g* for 30′ at 4°C, and then at 100000×*g* for 4 h to precipitate a pellet containing Aβ oligomers and fibrils. The pellet was solubilized in 70% formic acid for 1 h at room temperature, concentrated under vacuum and diluited 1∶10 in 2 M Trizma and then 1∶3 in H_2_O before assessing protein concentration by BCA Protein Assay Kit (Pierce) and performing the Aβ_1-42_ quantification by ELISA assay.

Aβ_1-42_ levels were measured in duplicates using a well established sandwich ELISA kit (β-Amyloid-42 ELISA kit High-Sensitive, Wako) containing two highly specific antibodies for detection of the Aβ_1-42_ peptide, according to manufacturer's instructions.

### Statistical Analyses

Unpaired data were analyzed with Student's *t* test, and *p* values of <0.05 and <0.01 were considered significant. Data are presented as means ± S.D.

## Results

### APP processing in HSV-1-infected neurons yields multiple APP fragments

**Figure 1:**
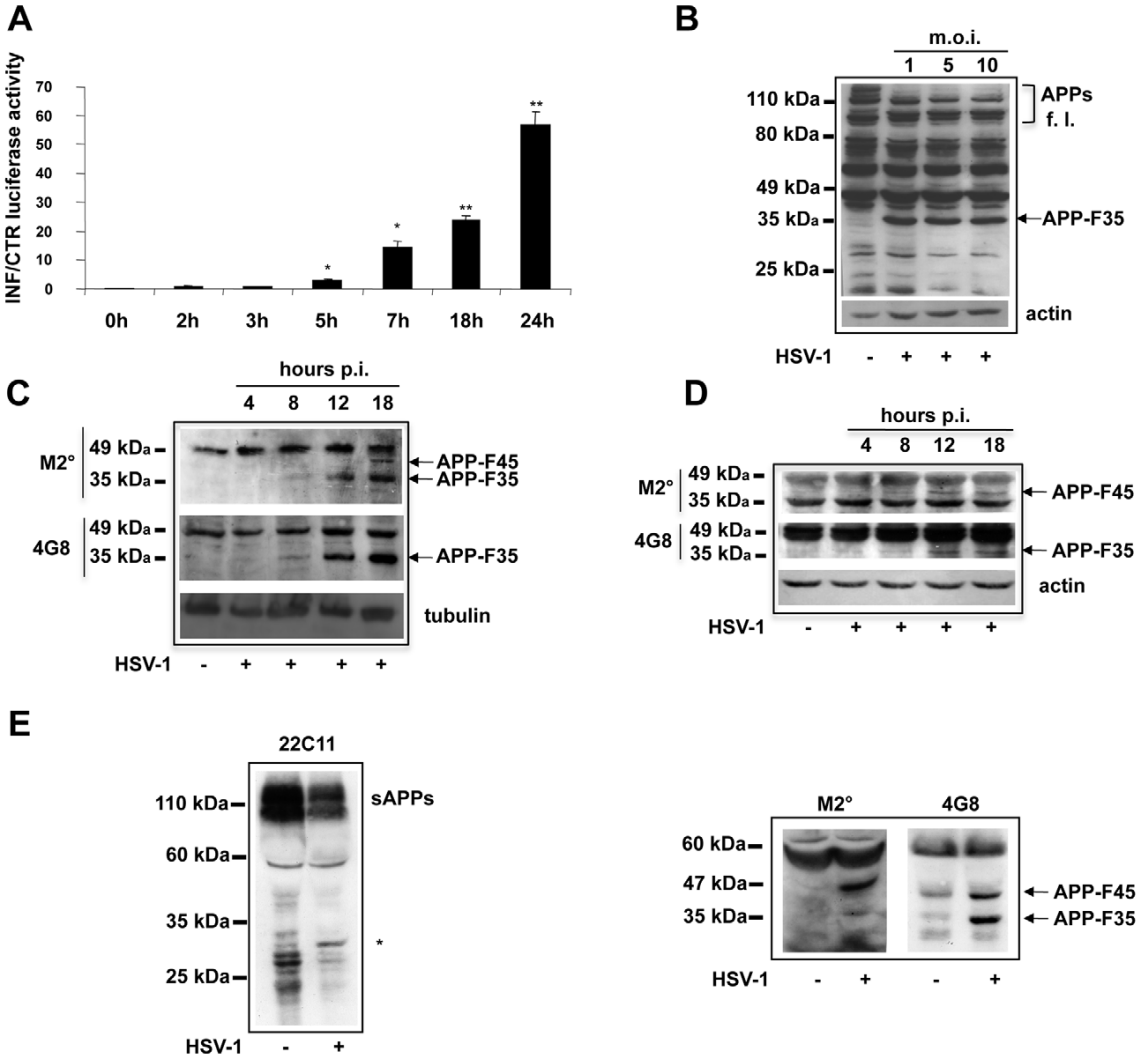
HSV-1 alters APP processing in neuronal cells promoting the formation of 35- and 45-kDa fragments. (A) Luciferase assay in HeLa cells that had been stably transfected with an APP-Gal4 fusion protein, transiently co-transfected with the G5B-luciferase vector, and infected with HSV-1 (m.o.i. 1). Luciferase activity was measured as an index of APP cleavage at different times p.i.. Data are shown as ratios of values measured in infected cell lysates (INF) to those in control cell lysates (CTR). Each bar represents the mean ratio ± S.D. (n = 6) of 3 individual experiments, each performed in duplicate. *p<0.05 and **p<0.01 vs. 0 h p.i.. (B) Western blot analysis of APP processing in SH-SY5Y cells infected with HSV-1 at different m.o.i. (1, 5, and 10) and harvested 18 h p.i. Blots were probed with 4G8 antibody. The membrane was then stripped and reprobed with anti-actin antibody. Bands representing full-length APPs and APP-F35 are indicated. (C) Western blot analysis of the time course of APP processing in SH-SY5Y cells after HSV-1 infection (m.o.i. 1). The membrane was probed with M2° and then stripped and reprobed with 4G8. Tubulin was used as a loading control. Arrows show bands representing APP-F35 and another APP fragment weighing 45 kDa (APP-F45). (D) Rat cortical neurons were infected and subjected to the same analysis described in C. (E) TCA-precipitated proteins from the supernatants of HSV-1-infected SH-SY5Y cells (showed in figure 1C, 18 h p.i.) were analyzed by western blot with an anti-N-terminal APP antibody (22C11, MAB348) (left panel) and with M2° and 4G8 antibodies (right panels). Released soluble α- and β-APPs (sAPPs), APP-F35 and APP-F45 are indicated. The star in the left panel shows an unidentified 30-kDa N-terminal APP fragment. Results are shown for one representative experiment of three performed.

To investigate the effects of HSV-1 infection on APP processing, we first used a luciferase reporting system, based on a recombinant protein (APP-Gal4) obtained by fusing the yeast transcription factor Gal4 to the C terminus of APP695, the main APP isoform in the human brain. HeLa cells were stably transfected with a vector encoding APP-Gal4 (HeLaAG cells) and then transiently co-transfected with a G5B-luciferase vector, in which luciferase expression is under the control of 5 Gal4-responsive elements. Luciferase activity was measured as a readout of APP cleavage [37]. Twenty-four hours after transfection, the HeLaAG cells were infected with HSV-1 at an m.o.i. of 1. Measurement of luciferase activity in cells harvested at different times post-infection (p.i.) revealed time-dependent increases in APP-Gal4 cleavage (Figure 1A).

To characterize this postinfection APP processing in neuronal cells, we infected human neuroblastoma cells (SH-SY5Y) and primary rat cortical neurons and monitored APP cleavage by western blot analysis of cell lysates with a panel of antibodies directed against different epitopes of the Aβ domain. The first antibody we used, 4G8, recognizes residues 17–24 of Aβ. As expected, blots from mock-infected cells contained various bands corresponding to the 3 main isoforms of full-length APP in multiple glycosylation states [32] (Figure 1B). Some of these bands were markedly less intense in cells infected with HSV-1. In particular, the band presumably representing the mature form of APP695 was almost undetectable in blots from all infected cells, including those challenged with an m.o.i. of 1. The diminished presence of APP following HSV-1 infection is likely due to virus-induced suppression of cellular protein synthesis [39], but it could also reflect viral stimulation of APP processing. The latter possibility is consistent with the results of our luciferase assays, and it was confirmed by the fact that, in addition to several 4G8-immunoreactive APP fragments (APP-Fs) that were detected in both mock-infected cells and HSV-1 infected cells, a fragment of approximately 35 kDa (APP-F35) was found exclusively in cells that had been infected with HSV-1 (Figure 1B).

The kinetics of the APP-F35 production were investigated by western blot analysis of lysates of infected SH-SY5Y cells or cortical neurons that had been harvested at different times p.i. (Figures 1C and 1D). In these experiments, we also stained these blots with the M2° antibody produced by our group, which recognizes residues 1–10 of the Aβ domain. 4G8 labeling detected APP-F35 starting from 8 h p.i. in SH-SY5Y (Figure 1C), and from 12 h p.i. in cortical neurons (Figure 1D), and its accumulation in both cell types increased with time. This same band was also labeled by the M2° antibody in blots of infected SH-SY5Y cells. M2° labeling also revealed a 45-kDa APP fragment (APP-F45) that was not recognized by the 4G8 antibody, and its presence was evident in both types of cells following HSV-1 infection (Figures 1C and 1D). Neither of these APP-Fs was recognized by the MAB348 (clone 22C11) and MAB343 antibodies, which target the N- and C-terminals of APP respectively, so they do not appear to be APP-cleavage end-products (Figure S1, panel A). No cross-reactivity was found between the anti-HSV-1 antibodies and APP-F35 or APP-F45 or between the anti-Aβ antibodies and viral proteins from our HSV-1 preparations, which suggests that the 35-kDa and 45-kDa fragments are indeed generated by APP processing (Figure S1, panel B). Similar results were obtained when retinoic acid-differentiated SH-SY5Y cells were infected with HSV-1 and when cells were infected with different HSV-1 strains (F, KOS, and a clinical HSV-1 isolate) (data not shown). Therefore, subsequent experiments were performed only on undifferentiated SH-SY5Y cells infected with HSV-1 at an m.o.i. of 1, and the results were assessed 18 h p.i. unless otherwise specified. It is noteworthy that at this time point, cell viability was not significantly affected by viral infection (Figure S1, Panel C).

To determine whether post-infection APP processing yielded fragments that were secreted into the extracellular space, we subjected TCA-precipitated proteins from cell-conditioned culture medium to western blot analysis. APP fragments were found in the supernatants of both control and infected cells when blots were probed with antibody against the N-terminus of APP (clone 22C11, which recognizes APP residues 66–81) (Figure 1E, left panel). They had a molecular mass of approximately 100 kDa and may represent the secreted APP species produced by α- or β-secretase cleavage of APP at the level of the plasma membrane. Interestingly, secreted APP levels in medium from HSV-1-infected cell cultures appeared lower than those from control cultures, suggesting that their production diminishes after infection. This could be the result of virus-induced reductions in the expression of full-length APP or a shift to an alternative APP processing pathway triggered specifically by HSV-1 infection (see Figure 1). The supernatant of infected cells also contained a peptide of approximately 30 kDa that was recognized by the anti-N-terminal antibody. It may represent an additional N-terminal fragment generated by virally altered APP cleavage. Interestingly, both the 4G8 and M2° antibodies recognized APP-F35 and APP-F45 in the supernatants from infected cells (Figure 1E, right panels), suggesting that these APP-Fs are in part secreted. A similar pattern of APP-F secretion was also found at earlier time-points (12 h p.i.) (Figure S1, Panel C), thus indicating that the HSV-1-induced APP processing we observed is not simply an artifact due to the late infection time. Collectively, these results demonstrate that HSV-1 infection alters APP processing in neuronal cells and that this effect favors the formation of at least 2 APP-Fs that include portions of the Aβ sequence. Our subsequent experiments focused mainly on the intracellular APP-Fs produced after HSV-1 infection because a growing body of evidence is highlighting the pathological effects of intraneuronal accumulations of Aβ peptides (reviewed in [24]).

### Host-cell protein synthesis spared by the virus-induced shut-down is necessary for the production of APP-F35 and APP-F45

**Figure 2:**
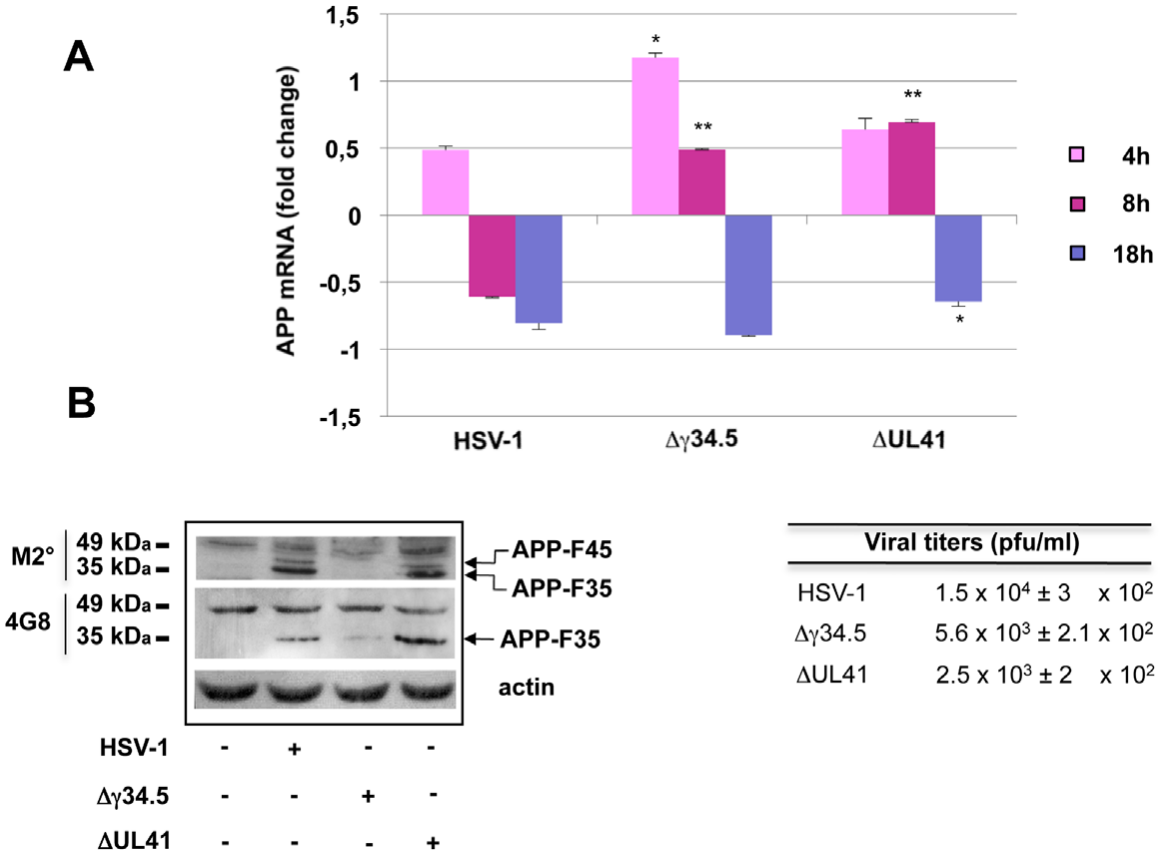
Host-cell protein synthesis spared by the virus-induced shut-down is necessary for APP-F35 and APP-F45 production. (A) Real-time PCR assay of APP mRNA levels in SH-SY5Y cells 4, 8 and 18 h after infection with wild-type or mutant (Δγ34.5 and ΔUL41) HSV-1 (m.o.i. 1). APP mRNA levels are expressed as fold changes versus mock-infected cells. At each indicated time point, data are shown as means ± S.D. of 4 independent experiments. For each time *p<0.05 and **p<0.01 vs HSV-1. (B) SH-SY5Y cells were infected with wild-type or mutant (Δγ34.5 and ΔUL41) HSV-1 for 18 h. Extracted proteins were subjected to SDS-PAGE, blotted and probed with M2°, 4G8, and anti-actin antibodies (left). Results are shown for one representative experiment of four performed. Conditioned medium samples were subjected to standard plaque assay to evaluate viral production (right). Data represent the mean ± S.D. of 8 independent experiments, each performed in duplicate.

As shown in Figure 1B, HSV-1 infection diminished the amount of full-length APP. Virus-induced shut-down of host cell protein synthesis, which is mediated primarily by the degradation of host protein mRNA [39], mainly by the virion host shut-off (vhs) protein [40], seemed to be at least partly responsible for this decrease. Indeed, real-time PCR assays revealed that in infected SH-SY5Y cells APP transcript levels progressively decreased starting from 4 h p.i. up to 18 h p.i. (Figure 2A). Comparable reductions were observed in infected cortical neurons assayed at 18 h p.i. (Figure S2A). The suppression of host protein synthesis can affect the expression of various genes [41], but efficient translation of certain viral and cellular proteins is essential to allow the virus to complete its life-cycle. For this reason, HSV-1 has evolved mechanisms for partial reversal of the shut-off. One of these involves reactivation of cellular translation by ICP34.5, the protein encoded by the *UL34.5* gene [42].

Consistently, when cells were infected with the HSV-1 R2621 mutant (hereafter referred to as ΔUL41), which is deleted in the *vhs* gene, APP mRNA levels were significantly increased over that observed in cells infected with wild-type HSV-1, although it was still lower than that of control cells 18 h p.i., since *vhs* is not the only viral gene involved in the degradation of host-cell mRNA (Figure 2A). On the other hand, when cells were infected with an HSV-1 mutant (R3616, referred to hereafter as Δγ34.5) that is deleted in both copies of the *UL34.5* gene, APP mRNA accumulated in the early phases of infection (4 h p.i.) probably as a consequence of the impaired translation. Later, APP mRNA levels were progressively decreased likely because of degradation of untraslated mRNAs (Figure 2A). Both mutants were able to infect SH-SY5Y cells efficiently, as shown by viral titers in the supernatant (see Table in Figure 2B).

Western blotting was then used to investigate APP-F formation in cells infected with the HSV-1 mutants. We reasoned that if the presence of the APP-F35 and APP-F45 in infected cells reflected viral downregulation of the expression of one or more catabolic enzymes involved in the degradation of APP fragments, higher levels of APP-F35 and APP-F45 would be found in Δγ34.5-infected cells, where the shut-off of protein synthesis is almost complete. In contrast, if the formation of these fragments required the synthesis of specific enzyme(s), they would not be found at all in Δγ34.5-infected cells. As shown in Figure 2B, the latter hypothesis was confirmed: APP-F35 and APP-F45 were clearly present in cells infected with wild-type HSV-1 or the ΔUL41 mutant, but they were almost undetectable in Δγ34.5-infected cells. Stronger inhibition of protein synthesis thus appears to impede rather than promote the formation of these fragments.

Accordingly, when cells were infected for 18 h in the presence of cycloheximide (CHX), a potent inhibitor of protein synthesis, APP-F formation was totally prevented (Figure S2B). The same effect was obtained when cells were infected with heat- or UV-inactivated viruses, unable to penetrate into host cells or to replicate, respectively (Figure S2C). Finally, APP-F formation was strongly inhibited when cells were infected in the presence of phosphonacetic acid (PAA) that blocks viral DNA synthesis and, as a consequence, late viral protein (γ) production (Figure S2B). Overall these findings support the statement that both viral replication and active cellular protein synthesis are required for APP-F formation.

### β- and γ-secretases and caspases are involved in the formation of APP-F35 but not that of APP-F45

**Figure 3:**
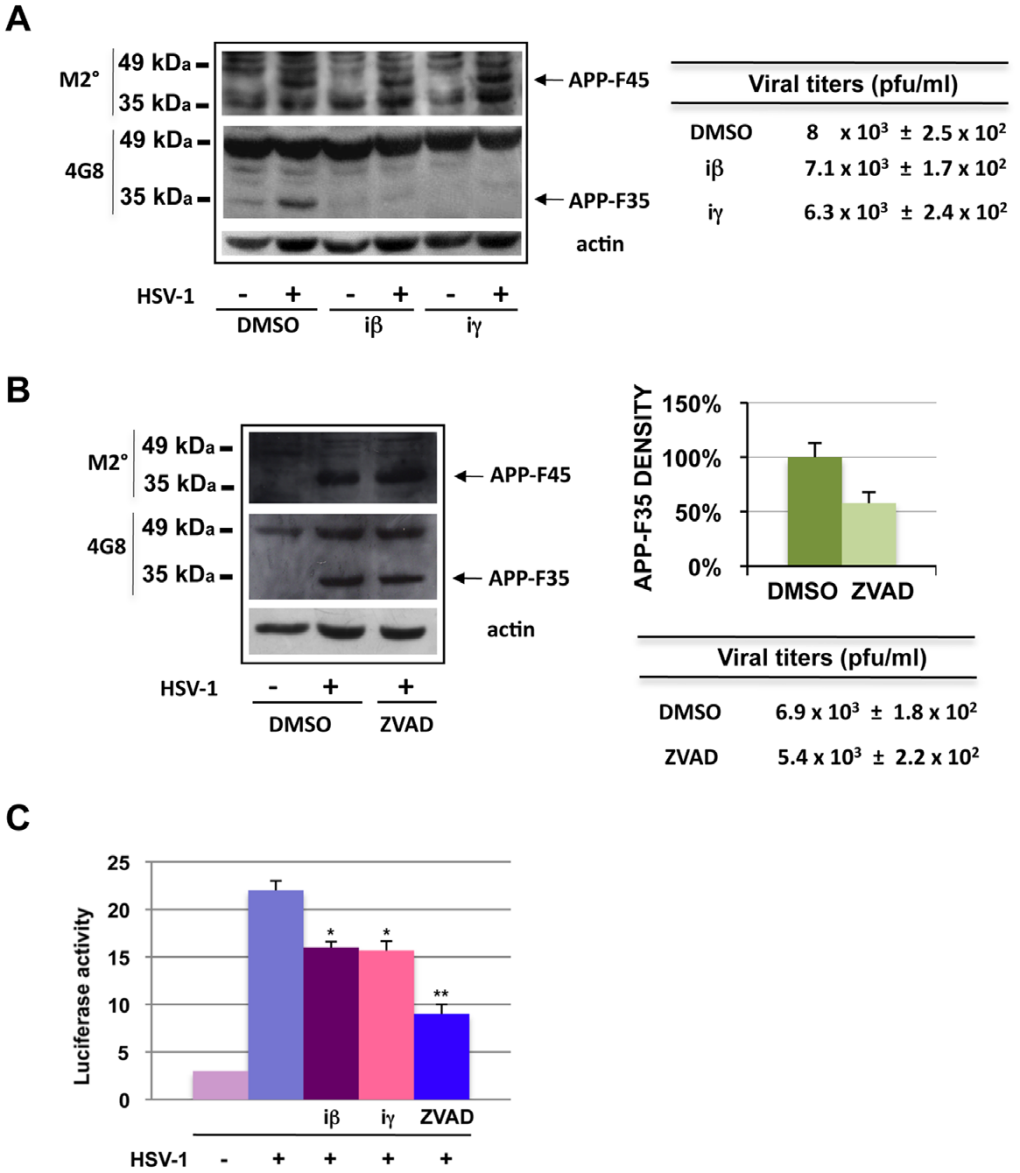
β- and γ-secretase are involved in APP-F35 formation. (A) HSV-1-infected (m.o.i. 1) and mock-infected SH-SY5Y cells were treated continuously (1 hour before infection through p.i. hour 18) with 1 µM of a β- or γ-secretase inhibitors (iβ and iγ, respectively). Control cultures (infected and mock-infected) were treated for the same period with equal volumes of solvent (DMSO). Cell lysates were analyzed by western blot with M2° and 4G8 antibodies. Actin was used as loading control. Results are shown for one representative experiment of three performed. Viral production estimated by standard plaque assay is shown next to the western blot. Data are means ± S.D. of 4 independent experiments. (B) Similar experiments were performed independently (as described in A) with 50 µM Z-VAD, which inhibits caspase 3-like enzymes. Densitometric analysis of APP-F35 levels is shown in the graph next to the representative western blot (Z-VAD-treated vs DMSO-treated HSV-1-infected cells) and data are the means ± S.D. of 3 independent experiments performed. Viral production estimated by standard plaque assay is shown. (C) HeLaAG cells were transfected with G5B-Luciferase vector and 24 h later infected with HSV-1 (m.o.i. 1) in the presence of an inhibitor of β-secretase, γ- secretase (1 µM each), or caspase 3-like enzymes (50 µM). Cells were harvested 18 h later and assayed for luciferase activity as a readout of APP cleavage. Data are the means ± S.D. of 3 independent experiments, each performed in duplicate. *p<0.05 and **p<0.01 vs. HSV-1.

The results of the previous experiments prompted us to investigate the involvement of cellular enzymes that normally cleave the APP in the altered APP processing observed in HSV-1 infected cells. SH-SY5Y cells were challenged with the virus in the presence of β-secretase inhibitor, γ-secretase inhibitor X, or Z-VAD, which specifically inhibits caspase-3-like enzymes. Control cells were HSV-1- or mock-infected in the presence of DMSO alone. Cells were harvested 18 h p.i. and subjected to western blot analysis with 4G8 and M2° antibodies. As shown in Figure 3, formation of APP-F35 decreased markedly in the presence of each of the secretase inhibitors (Figure 3A) and to a lesser extent when caspase-3-like activity was suppressed (Figure 3B). APP-F45 levels were not affected by any of the 3 inhibitors (Figure 3A and 3B). None of the inhibitors significantly altered the viral titers in the supernatant (also shown in Figure 3), so the effects of the secretase inhibition on APP-F35 cannot be attributed to interference with viral replication. We also found that expression levels of both BACE1 and nicastrin (an essential component of the γ-secretase complex) increased after HSV-1 infection (Figure S3, Panel A), suggesting that the virus upregulates their expression to drive amyloidogenic APP processing. Taken together, these findings point to major roles for β- and γ-secretases in APP-F35 formation, but neither of these enzymes is involved in the generation of APP-F45.

To explore the possibility that virus-induced oxidative stress plays a role in APP processing, we infected SH-SY5Y cells in the presence of two different antioxidant compounds (6-hydroxy-2,5,7,8-tetramethylchroman-2-carboxylic acid [TROLOX] and N-acetyl-L-cysteine [NAC]). As shown in Figure S3, Panel B, the results demonstrate that oxidative stress is mainly involved in APP-F35 formation. As expected, antioxidants did not significantly alter supernatant viral titers (Figure S3, Panel B), so their inhibitory effects on APP-F formation cannot be attributed to interference with viral replication.

However, APP-F35 and APP-F45 might not be the only APP fragments generated by APP processing in HSV-1-infected cells. Therefore, we used the luciferase system described above to detect all C-terminal APP-Gal4 fragments produced following HSV-1 infection. Twenty-four hours after transfection with the G5B-Luciferase vector, HeLaAG cells were infected with HSV-1 (m.o.i. 1) in the presence of β-secretase inhibitor, γ-secretase inhibitor X, Z-VAD, or (in controls) DMSO, and luciferase activity was measured 18 h p.i.. As shown in Figure 3C, APP cleavage in infected cells was slightly but significantly reduced by β- or γ-secretase inhibition. This result, which is in line with the findings shown in Figure 3A, confirms that these cellular enzymes are responsible for some, but not all, the APP cuts induced by the infection. Z-VAD appeared to be the most efficient suppressor of post infection APP cleavage in the luciferase assay (Figure 3C). This was not surprising since caspase-3-like enzymes cleave APP at its C-terminus, and C-terminal APP-Gal4 fragments are the ones detected by our luciferase assay.

Collectively, these data suggest that in the presence of HSV-1 APP undergoes cleavage at multiple sites. Some of the cuts seem to be produced by caspase-3-like enzymes; others (in particular those that generate APP-F45) seem to be produced by other cellular or viral enzymes that have yet to be identified. Other cleavages -like those that generate APP-F35- are produced by the same enzymes responsible for the formation of Aβ. These were the focus of the remaining experiments.

### APP processing in HSV-1-infected neurons yields a soluble Aβ oligomer (APP-F35) and Aβ_1-40_ and Aβ_1-42_ peptides

**Figure 4:**
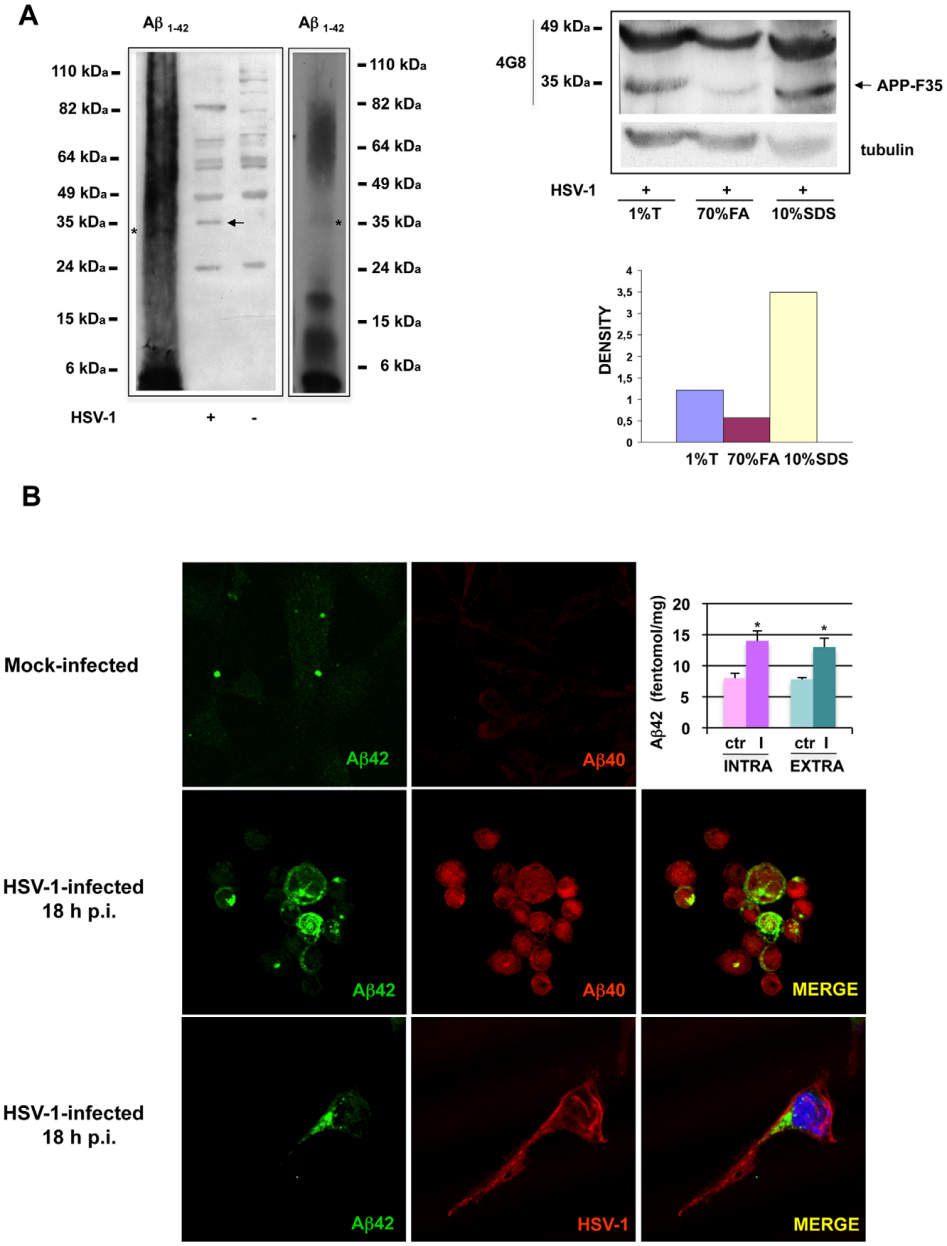
APP-F35 is a soluble oligomer of Aβ peptides. (A) A pool synthetic Aβ_1-42_ oligomers and lysates of mock-infected and HSV-1 infected cells were subjected to SDS-PAGE and western blot analysis with 4G8 antibody. A better visualization of the Aβ_1-42_ oligomeric mixture is provided in the western blot on the right (few seconds of exposure time). The stars indicate the Aβ nonamer whose electrophoretic mobility is similar to that of APP-F35, indicated with the arrow (left panel). HSV-1-infected cells were lysed with 1% triton-X 100, 70% formic acid, or 10% SDS. The samples were resolved by SDS-PAGE, blotted, and immunostained with 4G8 antibody. The membrane was stripped and restained with anti-tubulin antibody (loading control). Western blot is shown for 1 representative experiment of 3 performed (upper right panel). Immunoblots were analyzed densitometrically, and the values were expressed as ratios of APP-F35 to actin (lower right panel). (B) Confocal microscopic images of SH-SY5Y cells 18 h after infection with HSV-1 (m.o.i. 1). Cells were double-labeled with anti-Aβ_1-40_ and anti-Aβ_1-42_ antibodies (middle panels) or with anti-Aβ_1-42_ and anti-HSV-1 antibodies (lower panels). The color of the fluorescence representing each primary antibody is indicated. Results are shown for one representative experiment of three performed. Quantitation of Aβ_1-42_ from mock- and HSV-1-infected APP695-transfected SH-SY5Y cells by ELISA is shown (upper right). Bar graphs represent the levels (fentomol/mg) of intracellular (pellet) and secreted (medium) Aβ_1-42_. Data are the means ± S.D. of 3 independent experiments, each performed in duplicate. * p<0.05 vs. HSV-1.

The results of experiments shown in Figure 3A indicate that the formation of APP-F35 requires the activity of the same enzymes (β- and γ-secretases) that are responsible for the generation of Aβ. Since the molecular weight of APP-F35 is approximately 9-fold higher than that of Aβ (4 kDa), we wondered whether APP-F35 might be one of the SDS-stable Aβ oligomers whose formation has been associated with memory deficits in an experimental model of AD [43]. This hypothesis was compatible with the electrophoretic mobility of APP-F35, which was similar to that of the nonameric component of a mixture of oligomers of synthetic Aβ_1-42_ peptides (Figure 4A, left panel). To test this hypothesis, we subjected infected SH-SY5Y cells to lysis with three different protocols: 1% triton X-100 (the usual protocol), 10% SDS (which causes partial solubilization of aggregates), and 70% formic acid (which is generally employed to dissolve Aβ aggregates) [44]. As shown in Figure 4A (right panel), the 4G8-immunoreactive 35-kDa band was present in western blots from 1% triton-X-100-lysed cells and from those solubilized in 10% SDS, but it was much less intense in blots from formic-acid-treated cells.

In order to quantify Aβ_1-42_ produced during HSV-1 infection, we performed an ELISA assay on solubilized Aβ oligomers isolated from cell lysates or supernatants of SH-SY5Y cells transiently transfected with a vector codyfing APP695. The graph in Figure 4B shows that both intra- and extra-cellular Aβ_1-42_ levels are significantly increased in infected cells as compared with controls. These findings and those reported in Figure 1E suggest that HSV-1 infection may induce the secretion of highly neurotoxic APP-Fs, including Aβ peptides. To document the neurotoxic potential of these fragments, we challenged primary rat cortical neurons with the supernatants of SH-SY5Y cells infected with HSV-1 (1 m.o.i., 18 h) in the presence or absence of β- and γ-secretase inhibitors and assessed the number of apoptotic neurons 24 h later. Rat cortical neurons appeared to be a suitable model for these tests. Indeed, exposure to the supernatants from infected cells triggered apoptosis in these neurons even when the supernatants were exposed to UV light to inactivate the virus they contained (Figure S4). Interestingly, the supernatants from SH-SY5Y cells infected in the presence of β- and γ-secretase inhibitors were much less neurotoxic. Collectively, these data suggest that supernatants from HSV-1-infected cells are highly neurotoxic for primary neurons, and that this effect is related to the presence in the extracellular medium of APP-Fs rather than of “active” viral particles.

Direct evidence of HSV-1-induced Aβ formation in native non-trasfected cells was obtained though immunofluorescence studies with antibodies that specifically recognize the Aβ_1-40_ and Aβ_1-42_ peptides that showed that both forms were clearly present in HSV-1 infected cells, but they were almost undetectable in mock-infected cells. Interestingly, the Aβ_1-40_ staining was mainly nuclear, while Aβ_1-42_ was predominantly perinuclear, which is consistent with previous observations under other experimental conditions [45].

**Figure 5:**
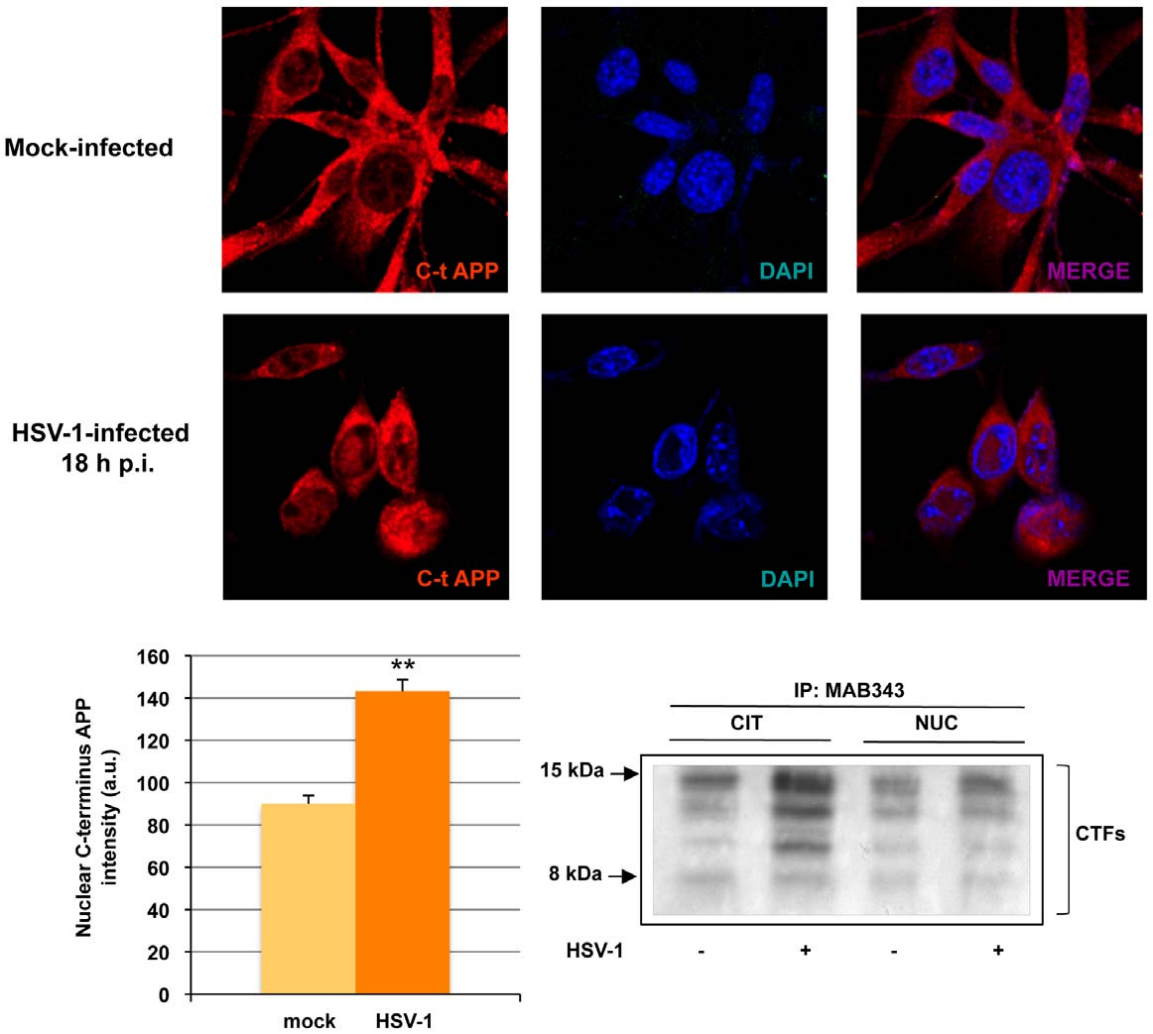
HSV-1 infection promotes nuclear localization of AICD. Confocal microscopic images of SH-SY5Y cells 18 h after mock or HSV-1 (m.o.i. 1) infection. Cells were labeled with anti-C-terminus-APP antibody (MAB343) and subjected to nuclear DAPI staining. Results are shown for one representative experiment of three performed. Bar graphs showing mean nuclear C-terminus APP labeling intensities in mock- and HSV-1-infected cells. **p<0.01 vs HSV-1 (n = 60). Nuclear and cytoplasmic extracts from mock- and HSV-1-infected cells were immunoprecipitated with anti-APP C-terminus antibody (MAB343) and the samples were resolved by SDS-PAGE. Western blot is one representative experiment of three performed.

The possibility of cross-reactivity between the anti-Aβ antibodies and the HSV-1 glycoprotein B was excluded by the results of double-staining with anti-HSV-1 and anti-Aβ_1-42_ antibodies (Figure 4B). The immunofluorescence was not colocalized with anti-Aβ_1-42_ antibody staining, confirming that this labeling actually reflected the virus-induced production of the amyloid peptides. We also labeled infected cells with MAB343, an anti-C-terminal-APP antibody that targets residues 640–695 of APP695. It recognizes full-length APP, the APP C-terminal fragments produced by α- and β-secretase cuts (CTFs), and the APP intracellular C-terminus domain (AICD) generated by γ-secretase cleavage of APP. The latter peptide is rapidly transported into the nuclear compartment, where it promotes the transcription of genes involved in neurodegeneration [18]. As expected, nuclear MAB343 immunolabeling was very faint in mock-infected cells but quite intense in cells infected with HSV-1 (Figure 5). This finding indicates that the virus enhances the nuclear translocation of the AICD, and it is consistent with the results of our luciferase assays, which demonstrated increased formation and nuclear localization of C-terminal APP fragments in infected HeLaAG cells (see Figure 1A). Interestingly, western blot analysis of CTFs immunoprecipitated from cytoplasm and nuclei of mock- infected or HSV-1-infected SH-SY5Y cells 18 h p.i. indicates that they increased in the cytoplasm and in the nuclei of infected cells, supporting data described so far. Taken together, these data clearly demonstrate that HSV-1 infection alters APP processing and that this effect results in the formation of a soluble oligomer of Aβ, Aβ_1-40_ and Aβ_1-42**,**_ and the increased presence of AICD and CTFs in the nucleus.

## Discussion

In this paper, we demonstrate that HSV-1 interferes with APP processing in SH-SY5Y human neuroblastoma cells and rat cortical neurons. Following HSV-1 infection, these cells were found to contain several APP-Fs that were not present in mock-infected control cells. These included 2 species containing at least parts of the Aβ domain of the protein (APP-F35 and APP-F45), N-terminal APP-Fs that were secreted, intracellular C-terminal fragments, and finally the Aβ_1-40_ and Aβ_1-42_ peptides. The HSV-1-triggered APP processing involved multiple cleavages that were mediated by different molecular mechanisms. Some (but not all) of the cuts were found to be produced by cellular enzymes that are known components of the amyloidogenic APP processing pathway (β- and γ-secretases, caspase-3-like enzymes).

Previous studies have revealed increased Aβ immunoreactivity in infected neuroblastoma and glioma cells and in the brains of HSV-1-infected mice [33], and accumulation of a 55-kDa C-terminal APP-F has been reported in HSV-1-infected SH-SY5Y cells [32]. This protein was clearly distinct from the APP-F35 and APP-F45 found in the western blots from our infected neuronal cells. Neither of these fragments was recognized by the anti-C terminal APP antibody used by the Shipley group or by antibody raised against the N-terminus of APP. However, C-terminal APP fragments were present in our infected cells, as shown by the results of our luciferase assays and immunofluorescence studies, and one of these may indeed be the protein observed by Shipley et al. The APP-F35 and APP-F45 found in our model both include at least parts of the Aβ sequence. The former fragment was recognized by M2° and 4G8 antibodies (which target Aβ residues 1–10 and 17–24, respectively), so it appears to include at least the first 24 residues of the Aβ peptide. The APP-F45 was recognized only by the M2° antibodies, suggesting that it contains only the first 10 Aβ residues.

Two species of approximately 35 kDa and 45 kDa were also recognized by both the M2° and 4G8 antibodies among the proteins precipitated from supernatants of infected cells. In all probability, these are the APP-F35 and APP-F45 that we detected in infected cell lysates. However, the intracellular APP-F45 was not recognized by the 4G8 antibody, whereas the 45 kDa species found in the supernatant was. This discrepancy might be explained by the presence of hidden epitopes in APP-F45 that were unmasked in the supernatant protein by the TCA precipitation step. On the other hand, it is also possible that the bands recognized in supernatant blots by 4G8 and M2° antibodies are distinct APP-Fs that were not found in the cell lysates because they are totally eliminated from the intracellular compartment by secretion. Additional confirmation of the ability of HSV-1 to alter APP processing was provided by the finding of a 30-kDa N-terminal APP-F in the supernatants from infected but not control cells.

Like others [32], we found that HSV-1 infection is followed by the disappearance of full-length APP in neuronal cell lysates. This phenomenon probably reflects the abnormal APP processing induced by the virus, but it is also related to the shut-down of host-protein synthesis. Involvement of the latter mechanism is supported by the markedly reduced levels of APP mRNA found in infected cells 18 h p.i. and by the increased transcript levels observed when cells were infected with the vhs-deficient HSV-1 mutant instead of wild-type HSV-1. However, when more complete suppression of protein synthesis was produced by infecting the cells with the Δγ34.5 mutant, which is incapable of reversing the host-induced shut-off of protein synthesis, APP-F35 and APP-F45 formation was almost completely abolished. The protein synthesis that continues after this shut-down thus appears to be essential to the APP cleavages induced by the virus. This finding, together with previous reports of increased BACE1 and nicastrin expression in HSV-1-infected SH-SY5Y cells [33], which was confirmed in our experimental model, suggests that the altered APP processing induced by the virus is carried out at least in part by cellular enzymes that are synthesized during infection. This conclusion is strongly supported by the marked reduction in APP-F35 formation during inhibition of β- or γ-secretase. The expression and activity of both these enzymes are known to be increased by oxidative stress conditions, like those detected by the presence of high levels of ROS and lipid peroxidation products [46]–[48]. Interestingly, results from *in vitro* and *in vivo* studies indicate that HSV-1 infection shifts the redox balance in host cells toward a pro-oxidant state [49]–[50]. Furthermore, in murine neuronal cells HSV-1 infection has been shown to increase the formation of ROS and products of lipid peroxidation [51], and high levels of lipid-peroxidation and protein-nitrosylation products have been detected in brain areas affected by acute or latent HSV-1 after infection at primary sites [52]–[53]. Together with our demonstration that antioxidant compounds prevent the virus-induced formation of APP-F35, these findings strongly suggest that HSV-1-induced oxidative stress in neuronal cells may activate β- and γ-secretases, thereby triggering APP processing and APP-F35.

β- and γ-secretases are the enzymes responsible for the production of Aβ peptides, but the APP-F35 is clearly a larger protein than either Aβ_1-40_ or Aβ_1-42_, (4 kDa). It might be an aggregate of Aβ peptides with other cellular or viral proteins, whose formation is induced by the viral infection. However, it might also represent an oligomeric form of Aβ. The latter conclusion is consistent with the infection-related overproduction of Aβ_1-40_ and Aβ_1-42_ documented by our immunocytochemistry experiments and with the electrophoretic mobility characteristics of APP-F35 itself. As shown in Figure 4A, this APP-F behaved like a soluble amyloid nonamer. The small difference between the apparent molecular weights of APP-F35 and the nonamer in the Figure probably reflects the origins of the two peptides, i.e., cell lysates vs. pooled synthetic peptides. These data were confirmed also by ELISA assay showing increased levels of Aβ_1-42_ in acid formic-solubilized cell lysates of infected cells.

Although cell enzymes clearly participate in the post-infection APP processing, the fact that the APP-F45 was unaffected by the β- and γ-secretase inhibitors strongly suggests that other mechanisms are also involved. Mapping studies indicate that APP-F45 contains only the first 10 residues of the Aβ domain, and it was not recognized by anti-APP N-terminal antibody (mapping sites 66–81). Species with such features might be seen during the degradation (i.e., elimination of the N-terminus) of a normal APP-F produced by α-secretase cleavage of APP at the level of an intracellular membrane (e.g., endoplasmic reticulum, Golgi apparatus). However, they would also be compatible with the presence of a novel APP fragment generated by the synergic actions of an α-secretase-like enzyme and another viral or cellular enzyme. Furthermore, experiments performed in the presence of the protein synthesis inhibitor CHX or PAA, a compound able to inhibit viral DNA synthesis, strongly confirm that APP-F45 formation is dependent on the occurrence of a correct protein synthesis and the production of late viral proteins. Among these, according to the data obtained in the experiments with Δγ34.5 mutant, ICP34.5 may be a potential candidate. Additional research is needed to clarify this point.

Other findings also support the hypothesis that HSV-1 induces multiple cleavages of APP, including some that are produced by caspase 3-like enzymes. Several studies have demonstrated caspase-mediated cleavage of APP between the Asp664 and Ala665 residues in its cytoplasmic domain although it is unclear whether caspases 3, 6, or 8 are primarily responsible for this cut. In any case, the result is a C-terminal fragment (C31) that promotes neurodegeneration by activating various cell-death pathways [54]. Some investigators have suggested that caspase cleavage may also promote subsequent cuts by γ-secretase, thereby shifting APP processing toward the amyloidogenic pathway. Our data support this view: APP-F35 formation was also partially suppressed by the caspase-3-like enzyme inhibitor Z-VAD, suggesting that in our experimental system β- and γ-secretase cleavages of APP are indeed increased by the protein's interaction with caspases. The presence of anti-C terminal-reactive APP fragments in the nucleus of infected cells might reflect nuclear transport of C31 and the AICD. AICD is also produced by γ-secretase cleavage of APP, and its role in triggering the transcription of genes involved in neurotoxicity, APP metabolism, and cytoskeletal dynamics has been well characterized [55]–[57]. In our experimental conditions, AICD was undetectable by western blot assay, whereas CTFs, probably derived from β-secretase cuts, are increased in the cytoplasm and in the nucleus of infected cells. Their presence in the nuclei suggests that they may play an additional role in triggering neurodegeneration with a still undisclosed mechanism.

In conclusion, our findings demonstrate that HSV-1 is capable of altering APP processing in neuronal cells and that this effect results in the formation of various species that are known to be neurotoxic. Indeed, we have clearly demonstrated that the APP fragments secreted by HSV-1-infected cells, especially those generated by β- and γ-secretase cleavages of the protein, can provoke apoptotic cell death in rat cortical neurons.

It is important to note that the cells used in our study had not been transfected to overexpress the APP. Therefore, the effects of infection we observed are probably a more faithful reflection of events that occur in vivo.

HSV-1 replication in the CNS can occur as a result of the reactivation of latent virus already present in the brain or of virus that has been reactivated in the trigeminal ganglia and reaches the brain by retrograde axonal transport. So far, the pathophysiological impact of APP fragments resulting from recurrent HSV-1 infection both in PNS and in CNS have not be described. However, a recent paper published by our group [34] clearly documented the functional alterations induced by HSV-1 infection in neurons, and data from Wozniak [33] evidenced the in vivo accumulation of Aβ plaques in brain of HSV-1-infected mice. It is possible to speculate that, while repeated cycles of replication would cause only mild and self-limiting infections, their repeated triggering of APP processing could cause intra- and extracellular accumulation of Aβ and the intranuclear transport of other neurotoxic APP fragments. Over long periods of time and in the presence of other genetic or environmental risk factors, these effects could play an important co-factorial role in the pathogenesis of AD.

## Supporting Information

Figure S1 APP-Fs do not contain APP-terminus domains and are not artifacts due to viral infection. (A) Lysates of mock- or HSV-1-infected SH-SY5Y cells were analyzed by western blot with 4G8, M2°, MAB343, and MAB348 (clone 22C11) antibodies. Bands corresponding to full-length APPs, APP-F35, and APP-F45 are indicated. (B) HSV-1 (F) was lysed in sample buffer and run on SDS-PAGE with HSV-1 (F)-infected neuroblastoma cell lysate (CL). The gel was blotted and the membrane probed with M2° and 4G8 antibodies. Bands corresponding to APP-F35 and APP-F45 are indicated by arrows in the HSV-1 lane. (C) TCA-precipitated proteins from the supernatants of HSV-1-infected SH-SY5Y cells (1 m.o.i., 12 h) were analyzed by western blot (left panel). APP-F35 and APP-F45 are indicated. Results are shown for one representative experiment of three performed. Cytotoxicity of HSV-1-infected SH-SY5Y cells (1 moi, 24 and 48 h) was determined by Trypan blue exclusion assay (right panel). Values are expressed as percentages of viable cells with respect to controls. All data represent the means ± S.D.
(0.82 MB TIF)

Figure S2 APP-F formation requires an active protein synthesis and the presence of late viral proteins (A) Real-time PCR assay of APP mRNA levels in rat cortical neurons harvested 18 h after infection with HSV-1 (m.o.i. 1). Data are shown as means ± S.D. of 3 independent experiments, **p<0.01 vs. mock-infected cells. (B) HSV-1-infected (m.o.i. 1) and mock-infected SH-SY5Y cells were treated continuously (1 hour before infection through p.i. hour 18) with 50 µg/ml of cycloheximide (an inhibitor of protein synthesis, CHX) and 500 µg/ml of phosphonoacetic acid (an inhibitor of the viral replication, PAA). Cell lysates were analyzed by western blot with M2° and 4G8 antibodies. Results are shown for one representative experiment of three performed. (C) SH-SY5Y cells were infected with HSV-1 or with heat- and UV-inactivated HSV-1 for 18 h. Extracted proteins were subjected to SDS-PAGE, blotted and probed with M2°, 4G8, and anti-actin antibodies. Results are shown for one representative experiment of four performed.
(0.68 MB TIF)

Figure S3 APP-F formation is partially inhibited by antioxidants (A) Lysates of mock- or HSV-1-infected SH-SY5Y cells were analyzed by western blot with anti-BACE1 (Chemicon) and anti-nicastrin (Millipore) antibodies. (B) Mock- and HSV-1-infected SH-SY5Y cells were treated after infection through p.i. hour 18 with 200 µM TROLOX or 5 mM N-Acetyl-L-Cysteine (NAC). Cell lysates were analyzed by western blot with M2° and 4G8 antibodies. Results are shown for one representative experiment of three performed. Densitometric analysis of APP-F35 and APP-F45 levels is shown in the graph next to the representative western blot (TROLOX- or NAC-treated HSV-1-infected cells with respect to HSV-1-infected cells). Viral production estimated by standard plaque assay is shown. Data are means ± S.D. of 3 independent experiments.
(0.33 MB TIF)

Figure S4 APP-F-containing supernatants from HSV-1-infected SH-SY5Y cells induce apoptosis in rat cortical neurons Apoptotic cell death was evaluated in rat cortical neurons by Vybrant® DyeCycle Violet Kit (Invitrogen) by using confocal laser scanning system (Leica TCS SP2). This assay is based upon a fluorescent dye (Vybrant® DyeCycle) able to stain chromatin. The condensed chromatin of apoptotic cells is stained more brightly than the chromatin of normal cells. Rat cortical neurons were challenged for 24 h with supernatants collected (18 h p.i.) from: (A) mock-infected cells (sup mock); (B) HSV-1-infected cells (sup HSV-1); (C) HSV-1-infected cells and then exposed to UV-light (5 min on ice) (sup HSV-1+ UV); (D) HSV-1-infected SH-SY5Y cells treated continuously (1 hour before infection through p.i. hour 18) with β- or γ-secretase inhibitors (sup HSV-1+iβ+iγ). Conditional medium derived from rat cortical neurons (cultured for 7 days) was used to culture SH-SY5Y cells for 18 h after HSV-1 challenge. Red arrows indicate representative apoptotic cells. The percentage of apoptotic cell death, shown in the graph, was evaluated on at least 10 microscopic fields randomly chosen for each conditions analyzed. Data are expressed as fold increase of apoptotic cells found in different experimental conditions versus controls (supernatants of mock-infected cells). ** P<0.01 vs sup HSV-1.
(2.11 MB TIF)

## References

[1] Schmutzhard E (2001). Viral infections of the CNS with special emphasis on herpes simplex infections.. J Neurol.

[2] Jamieson GA, Maitland NJ, Wilcock GK, Craske J, Itzhaki RF (1991). Latent herpes simplex virus type 1 in normal and Alzheimer’s disease brains.. J Med Virol.

[3] Itabashi S, Arai H, Matsui T, Higuchi S, Sasaki H (1997). Herpes simplex virus and risk of Alzheimer’s disease.. Lancet.

[4] Wozniak MA, Shipley SJ, Combrinck M, Wilcock GK, Itzhaki RF (2005). Productive herpes simplex virus in brain of elderly normal subjects and Alzheimer’s disease patients.. J Med Virol.

[5] Kastrukoff L, Hamada T, Schumacher U, Long C, Doherty PC (1982). Central nervous system infection and immune response in mice inoculated into the lip with herpes simplex virus type 1.. J Neuroimmunol.

[6] Dobson CB, Itzhaki RF (1999). Herpes Simplex virus type 1 and Alzheimer’s disease.. Neurobiol Aging.

[7] Lewandowski G, Zimmerman MN, Denk LL, Porter DD, Prince GA (2002). Herpes simplex type 1 infects and establishes latency in the brain and trigeminal ganglia during primary infection of the lip in cotton rats and mice.. Arch Virol.

[8] Ball MJ (1982). Limbic predilection in Alzheimer dementia: is reactivated herpes virus involved?. Can J Neurol Sci.

[9] Selkoe DJ (2001). Alzheimer’s disease: genes, proteins, and therapy.. Physiol Rev.

[10] Deatly AM, Haase AT, Fewster PH, Lewis E, Ball MJ (1990). Human herpes virus infections and Alzheimer's disease.. Neuropathol Appl Neurobiol.

[11] Jamieson GA, Maitland NJ, Craske J, Wilcock GK, Itzhaki RF (1991). Detection of herpes simplex virus type 1 DNA sequences in normal and Alzheimer’s disease brains using polymerase chain reaction.. Biochem Soc Trans.

[12] Hill JM, Gebhardt BM, Azcuy AM, Matthews KE, Lukiw WJ (2005). Can a herpes simplex virus type 1 neuroinvasive score be correlated to other risk factors in Alzheimer's disease?. Med Hypotheses.

[13] Mori I, Kimura Y, Naiki H, Matsubara R, Takeuchi T (2004). Reactivation of HSV-1 in the brain of patients with familial Alzheimer's disease.. J Med Virol.

[14] Itzhaki RF (2004). Herpes simplex virus type 1, apolipoprotein E and Alzheimer' disease.. Herpes.

[15] Letenneur L, Peres K, Fleuri H, Garrigue I, Barberger-Gateau P (2008). Sieropositive to herpes virus antibodies and risk of Alzheimer’s disease: a population-based cohort study.. Plos One.

[16] Sinha S, Anderson JP, Barbour R, Basi GS, Caccavello R (1999). Purification and cloning of amyloid precursor protein beta-secretase from human brain.. Nature.

[17] Vassar R (2001). The beta-secretase, BACE: a prime drug target for Alzheimer's disease.. J Mol Neurosci.

[18] Müller T, Meyer HE, Egensperger R, Marcus K (2008). The amyloid precursor protein intracellular domain (AICD) as modulator of gene expression, apoptosis, and cytoskeletal dynamics-relevance for Alzheimer's disease.. Prog Neurobiol.

[19] Chyung AS, Greenberg BD, Cook DG, Doms RW, Lee VM (1997). Novel beta-secretase cleavage of beta-amyloid precursor protein in the endoplasmic reticulum/intermediate compartment of NT2N cells.. J Cell Biol.

[20] Cook DG, Forman MS, Sung JC, Leight S, Kolson DL (1997). Alzheimer’s Abeta (1-42) is generated in the endoplasmic reticulum/intermediate compartment of NT2N cells.. Nat Med.

[21] Hartman T, Bieger SC, Bruhl B, Tienari PJ, Ida N (1997). Distinct sites of intracellular production for Alzheimer’s disease Abeta 40/42 amyloid peptides.. Nat Med.

[22] Xu H, Sweeney D, Wang R, Thinakaran C, Lo AC (1997). Generation of Alzheimer beta-amyloid protein in the trans-Golgi network in the apparent absence of vesicle formation.. Proc Natl Acad Sci USA.

[23] Koo EH, Squazzo SL (1994). Evidence that production and release of amyloid beta-protein involves the endocytic pathway.. J Biol Chem.

[24] LaFerla FM, Green KN, Oddo S (2007). Intracellular amyloid-beta in Alzheimer's disease.. Nat Rev Neurosci.

[25] Lu DC, Rabizadeh S, Chandra S, Shayya RF, Ellerby LM (2000). A second cytotoxic proteolytic peptide derived from amyloid beta-protein precursor.. Nat Med.

[26] Park SH, Shaked GM, Bredesen DE, Koo EH (2009). Mechanism of cytotoxicity mediated by the C31 fragment of amyloid precursor protein.. Biochem Biophys Res Comm.

[27] Cribbs DH, Azizeh BY, Cotman CW, LaFerla FM (2000). Fibril formation and neurotoxicity by a herpes simplex virus glycoprotein B fragment with homology to the Alzheimer's A beta peptide.. Biochemistry.

[28] Bearer EL (2004). Perspectives on herpes-APP interactions.. Aging cell.

[29] Satpute-Krishnan P, De Giorgis JA, Bearer EL (2003). Fast anterograde transport of herpes simplex virus: role for the amyloid precursor protein of Alzheimer disease.. Aging cell.

[30] Satpute-Krishnan P, De Giorgis JA, Conley MP, Jang M, Bearer EL (2006). A peptide zipcode sufficient for anterograde transport within amyloid precursor protein.. Proc Natl Acad Sci USA.

[31] Wozniak MA, Mee AP, Itzhaki RF (2009). Herpes simplex virus type 1 DNA is located within Alzheimer’s disease amyloid plaques.. J Pathol.

[32] Shipley SJ, Parkin ET, Ithzaki RF, Dobson CB (2005). Herpes simplex virus interferes with amyloid precursor protein processing.. BMC Microbiol.

[33] Wozniak MA, Itzhaki RF, Shipley SJ, Dobson CB (2007). Herpes simplex virus infection causes cellular -amyloid accumulation and secretase upregulation.. Neuroscience Letters.

[34] Piacentini R, Civitelli L, Ripoli C, Marcocci ME, De Chiara G (2010). HSV-1 promotes Ca2+-mediated APP phosphorylation and Aβ accumulation in rat cortical neurons.. Neurobiol Aging. In press.

[35] Fan YW, Cui FZ, Hou SP, Xu QY, Chen LN (2002). Culture of neural cells on silicon wafers with nano-scale surface topograph.. J Neurosci Methods.

[36] Killington RA, Powell KL, Mahy BWJ (1991). Growth, Assay and Purification of Herpesviruses.. Virology: a pratical approach.

[37] Gianni D, Zambrano N, Bimonte M, Minopoli G, Mercken L (2003). Platelet-derived growth factor induces the beta-gamma-secretase-mediated cleavage of alzheimer’s amyloid precursor protein through a Src-Rac-dependent pathway.. J Biol Chem.

[38] Piacentini R, Ripoli C, Mezzogori D, Azzera GB, Grassi C (2008). Extremely low-frequency electromagnetic fields promote in vitro neurogenesis via upregulation of Ca_v_1-channel activity.. J Cell Physiol.

[39] Matis J, Kudelova M (2001). Early shutoff of host protein synthesis in cells infected with herpes simplex viruses.. Acta Virol.

[40] Kwong AD, Frenkel N (1989). The herpes simplex virus virion host shutoff function.. J Virol.

[41] Roizmann B, Knipe DM, Knipe DM, Howley PM (2001). Herpes Simplex Virus and Their Replication.. Fields Virology.

[42] He B, Gross M, Roizman B (1998). The γ_1_34.5 protein of herpes simplex virus 1 has the structural and functional attributes of a protein phosphatase 1 regulatory subunit and is present in a high molecular weight complex with the enzyme in infected cells.. J Biol Chem.

[43] Lesné S, Koh MT, Kotilinek L, Kayed R, Glabe CG (2006). A specific amyloid-β protein assembly in the brain impairs memory.. Nature.

[44] Skovronsky DM, Doms RW, Lee VMY (1998). Detection of a novel intraneuronal pool of insoluble amyloid beta protein that accumulates with time in culture.. J Cell Biol.

[45] Ohyagi Y, Asahara H, Chui DH, Tsuruta Y, Sakae N (2005). Intracellular Abeta42 activates p53 promoter: a pathway to neurodegeneration in Alzheimer's disease.. FASEB J.

[46] Tamagno E, Bardini P, Obbili A, Vitali A, Borghi R (2002). Oxidative stress increases expression and activity of BACE in NT2 neurons.. Neurobiol Dis.

[47] Tamagno E, Parola M, Bardini P, Piccini A, Borghi R (2005). Beta-site APP cleaving enzyme up-regulation induced by 4-hydroxynonenal is mediated by stress-activated protein kinases pathways.. J Neurochem.

[48] Tamagno E, Guglielmotto M, Aragno M, Borghi R, Autelli R (2008). Oxidative stress activates a positive feedback between the gamma- and beta-secretase cleavages of the beta-amyloid precursor protein.. J Neurochem.

[49] Palamara AT, Perno CF, Ciriolo MR, Dini L, Balestra E (1995). Evidence for antiviral activity of glutathione: in vitro inhibition of herpes simplex virus type 1 replication.. Antiviral Res.

[50] Valyi-Nagy T, Dermody TS (2005). Role of oxidative damage in the pathogenesis of viral infections of the nervous system.. Histol Histopathol.

[51] Kavouras JH, Prandovszky E, Valyi-Nagy K, Kovacs SK, Tiwari V (2007). Herpes simplex virus type 1 infection induces oxidative stress and the release of bioactive lipid peroxidation by-products in mouse P19N neural cell cultures.. J Neurovirol.

[52] Fujii S, Akaike T, Maeda H (1999). Role of nitric oxide in pathogenesis of herpes simplex virus encephalitis in rats.. Virology.

[53] Valyi-Nagy T, Olson SJ, Valyi-Nagy K, Montine TJ, Dermody TS (2000). Herpes simplex virus type 1 latency in the murine nervous system is associated with oxidative damage to neurons.. Virology.

[54] Lu DC, Rabizadeh S, Chandra S, Shayya RF, Ellerby LM (2000). A second cytotoxic proteolytic peptide derived from amyloid beta-protein precursor.. Nat Med.

[55] Chang KA, Suh YH (2005). Pathophysiological roles of amyloidogenic carboxy-terminal fragments of the beta amyloid precursor protein in Alzheimer’s disease.. J Pharmacol Sci.

[56] Muller T, Concannon CG, Word MW, Walsh CN, Tirniceriu AL (2007). Modulation f gene expression and cytoskeletal dynamics by amyloid precursor protein intracellular domain (AICD).. Mol Biol Cell.

[57] Goodger ZV, Rajendran L, Trutzell A, Kohli BM, Nitsck RM (2009). Nuclear signalling by the APP intracellular domain occurs predominantly through the amyloidogenic processing pathway.. J Cell Sci.

