# Supplementary figures and images for: APP Processing Induced by Herpes Simplex Virus Type 1 (HSV-1) Yields Several APP Fragments in Human and Rat Neuronal Cells
Source: PLoS One. 2010 Nov 15;5(11):e13989. doi: 10.1371/journal.pone.0013989 (PMC2981559; doi:10.1371/journal.pone.0013989)

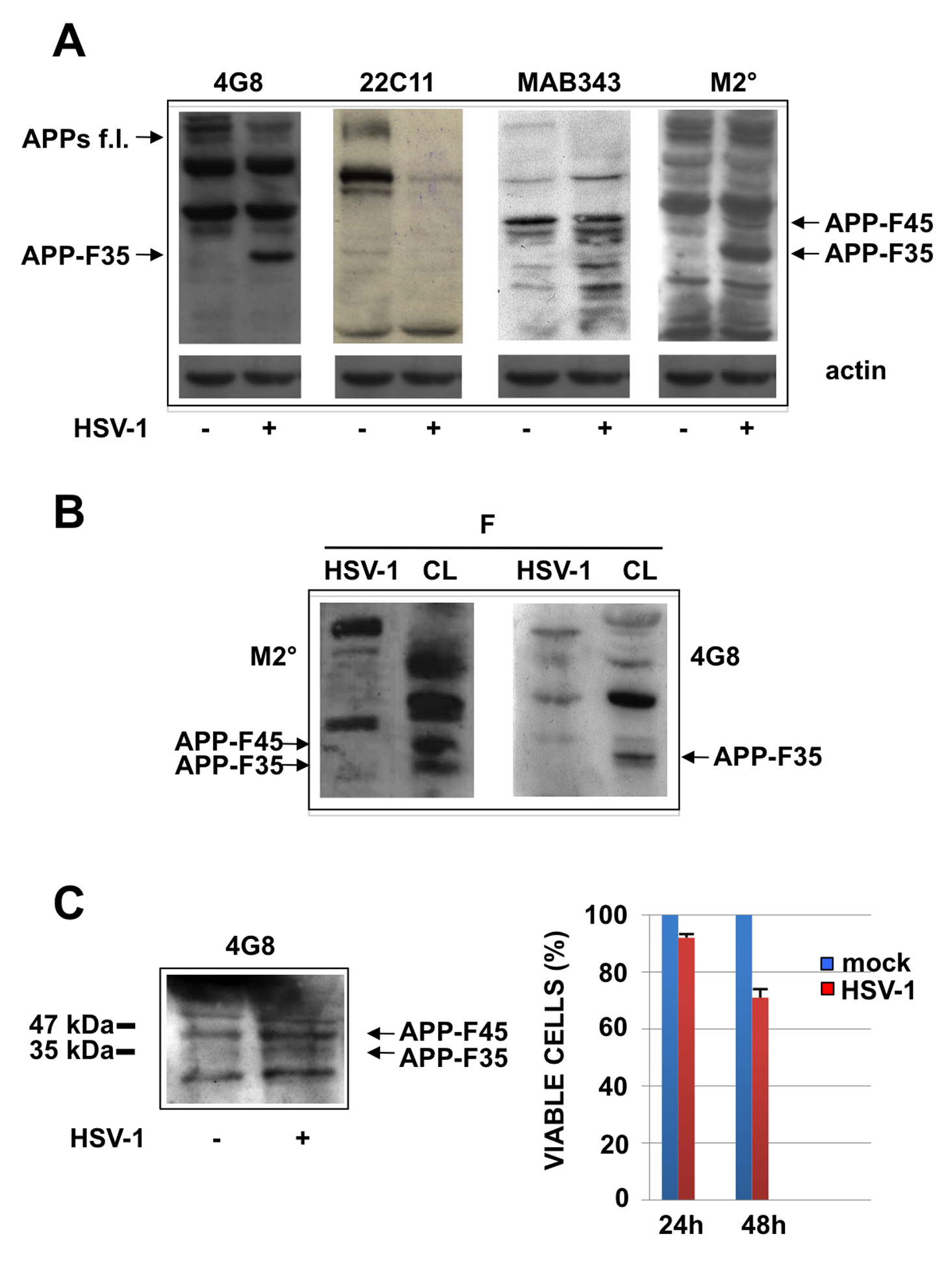

Supplement: Figure S1 — APP-Fs do not contain APP-terminus domains and are not artifacts due to viral infection. (A) Lysates of mock- or HSV-1-infected SH-SY5Y cells were analyzed by western blot with 4G8, M2°, MAB343, and MAB348 (clone 22C11) antibodies. Bands corresponding to full-length APPs, APP-F35, and APP-F45 are indicated. (B) HSV-1 (F) was lysed in sample buffer and run on SDS-PAGE with HSV-1 (F)-infected neuroblastoma cell lysate (CL). The gel was blotted and the membrane probed with M2° and 4G8 antibodies. Bands corresponding to APP-F35 and APP-F45 are indicated by arrows in the HSV-1 lane. (C) TCA-precipitated proteins from the supernatants of HSV-1-infected SH-SY5Y cells (1 m.o.i., 12 h) were analyzed by western blot (left panel). APP-F35 and APP-F45 are indicated. Results are shown for one representative experiment of three performed. Cytotoxicity of HSV-1-infected SH-SY5Y cells (1 moi, 24 and 48 h) was determined by Trypan blue exclusion assay (right panel). Values are expressed as percentages of viable cells with respect to controls. All data represent the means ± S.D. (0.82 MB TIF) [file pone.0013989.s001.tif]

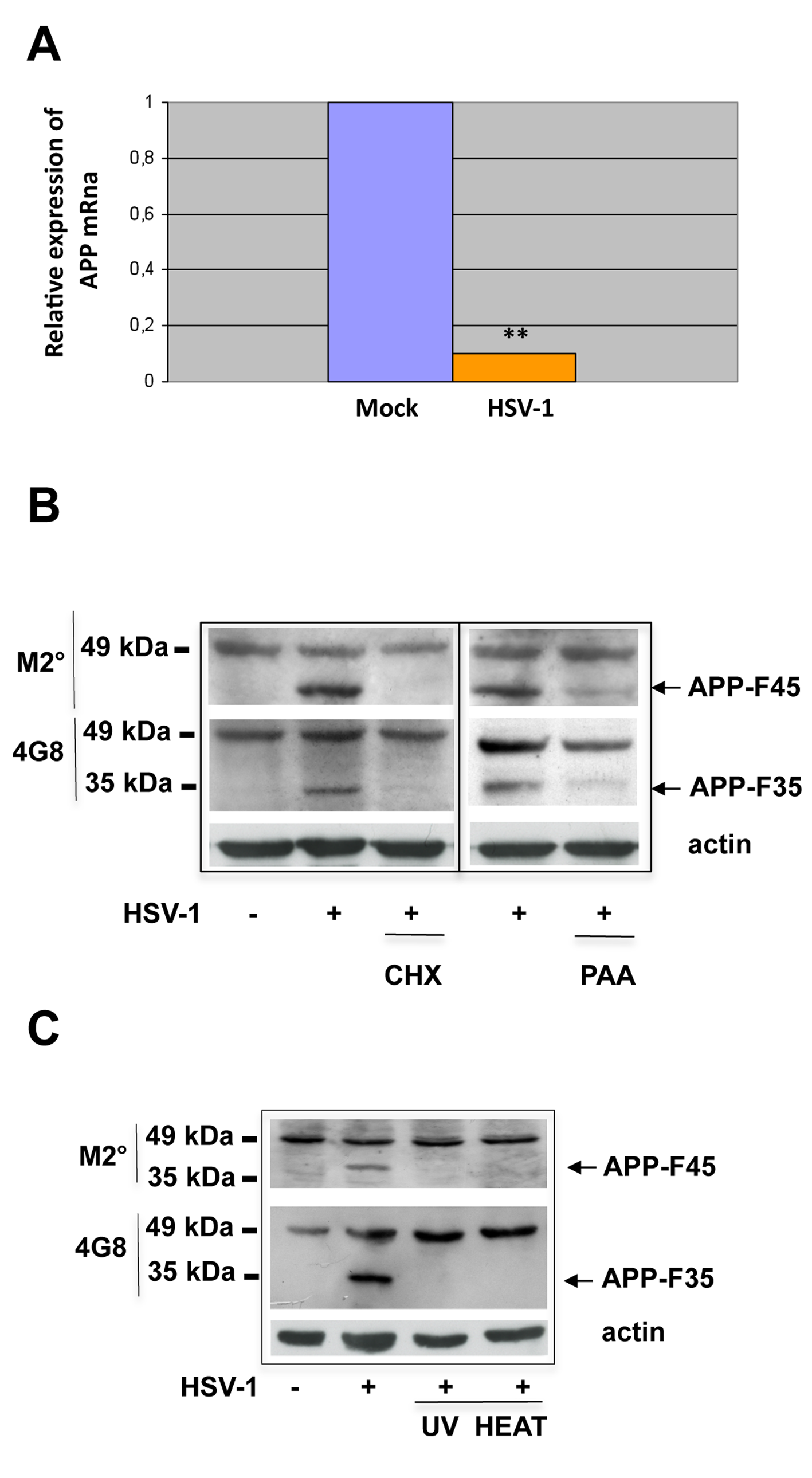

Supplement: Figure S2 — APP-F formation requires an active protein synthesis and the presence of late viral proteins (A) Real-time PCR assay of APP mRNA levels in rat cortical neurons harvested 18 h after infection with HSV-1 (m.o.i. 1). Data are shown as means ± S.D. of 3 independent experiments, **p<0.01 vs. mock-infected cells. (B) HSV-1-infected (m.o.i. 1) and mock-infected SH-SY5Y cells were treated continuously (1 hour before infection through p.i. hour 18) with 50 µg/ml of cycloheximide (an inhibitor of protein synthesis, CHX) and 500 µg/ml of phosphonoacetic acid (an inhibitor of the viral replication, PAA). Cell lysates were analyzed by western blot with M2° and 4G8 antibodies. Results are shown for one representative experiment of three performed. (C) SH-SY5Y cells were infected with HSV-1 or with heat- and UV-inactivated HSV-1 for 18 h. Extracted proteins were subjected to SDS-PAGE, blotted and probed with M2°, 4G8, and anti-actin antibodies. Results are shown for one representative experiment of four performed. (0.68 MB TIF) [file pone.0013989.s002.tif]

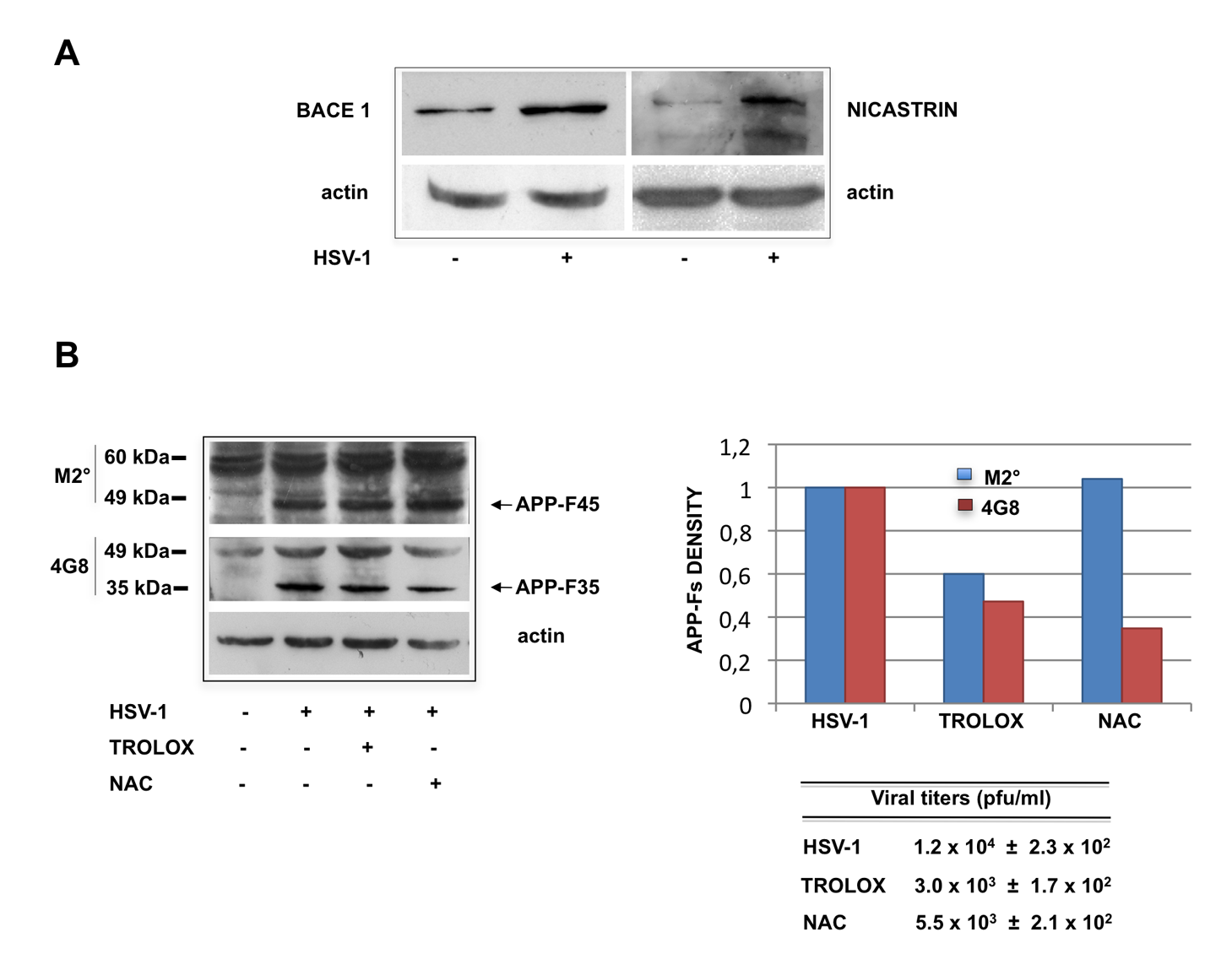

Supplement: Figure S3 — APP-F formation is partially inhibited by antioxidants (A) Lysates of mock- or HSV-1-infected SH-SY5Y cells were analyzed by western blot with anti-BACE1 (Chemicon) and anti-nicastrin (Millipore) antibodies. (B) Mock- and HSV-1-infected SH-SY5Y cells were treated after infection through p.i. hour 18 with 200 µM TROLOX or 5 mM N-Acetyl-L-Cysteine (NAC). Cell lysates were analyzed by western blot with M2° and 4G8 antibodies. Results are shown for one representative experiment of three performed. Densitometric analysis of APP-F35 and APP-F45 levels is shown in the graph next to the representative western blot (TROLOX- or NAC-treated HSV-1-infected cells with respect to HSV-1-infected cells). Viral production estimated by standard plaque assay is shown. Data are means ± S.D. of 3 independent experiments. (0.33 MB TIF) [file pone.0013989.s003.tif]

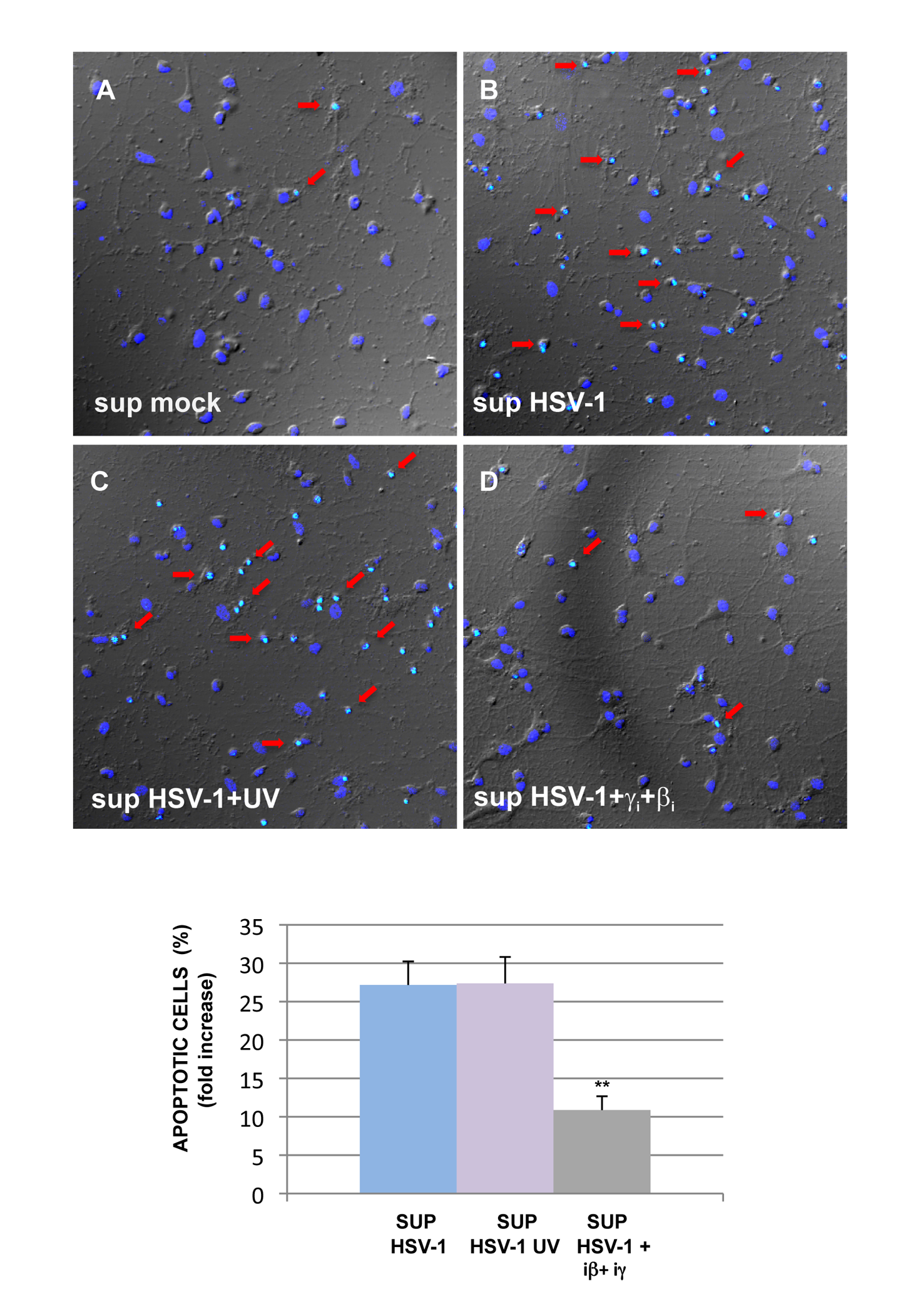

Supplement: Figure S4 — APP-F-containing supernatants from HSV-1-infected SH-SY5Y cells induce apoptosis in rat cortical neurons Apoptotic cell death was evaluated in rat cortical neurons by Vybrant® DyeCycle Violet Kit (Invitrogen) by using confocal laser scanning system (Leica TCS SP2). This assay is based upon a fluorescent dye (Vybrant® DyeCycle) able to stain chromatin. The condensed chromatin of apoptotic cells is stained more brightly than the chromatin of normal cells. Rat cortical neurons were challenged for 24 h with supernatants collected (18 h p.i.) from: (A) mock-infected cells (sup mock); (B) HSV-1-infected cells (sup HSV-1); (C) HSV-1-infected cells and then exposed to UV-light (5 min on ice) (sup HSV-1+ UV); (D) HSV-1-infected SH-SY5Y cells treated continuously (1 hour before infection through p.i. hour 18) with β- or γ-secretase inhibitors (sup HSV-1+iβ+iγ). Conditional medium derived from rat cortical neurons (cultured for 7 days) was used to culture SH-SY5Y cells for 18 h after HSV-1 challenge. Red arrows indicate representative apoptotic cells. The percentage of apoptotic cell death, shown in the graph, was evaluated on at least 10 microscopic fields randomly chosen for each conditions analyzed. Data are expressed as fold increase of apoptotic cells found in different experimental conditions versus controls (supernatants of mock-infected cells). ** P<0.01 vs sup HSV-1. (2.11 MB TIF) [file pone.0013989.s004.tif]
